# Eugenol preserves chondrocyte extracellular matrix homeostasis by modulating the ALK1/ALK5-associated TGF-β/Smad signaling axis

**DOI:** 10.3389/fphar.2026.1885138

**Published:** 2026-08-27

**Authors:** Yongcan Wang, Ying Wang, Shan Li, Xi Chen, Yaopu Hou, Chao Wang, Yanzhuo Zhang, Jianfeng Tao, Chengai Wu, Xu Jiang

**Affiliations:** 1 Department of Orthopaedics, National Center for Orthopaedics, Beijing Jishuitan Hospital, Capital Medical University, Beijing, China; 2 Department of Molecular Orthopaedics, National Center for Orthopaedics, Beijing Research Institute of Traumatology and Orthopaedics, Beijing Jishuitan Hospital, Capital Medical University, Beijing, China

**Keywords:** ALK1, ALK5, chondrocyte, eugenol, extracellular matrix, osteoarthritis, SMAD signaling, TGF-β signaling

## Abstract

Osteoarthritis (OA) is characterized by inflammatory and catabolic disruption of cartilage extracellular matrix (ECM) homeostasis. An increased relative abundance of activin receptor-like kinase 1 (ALK1) relative to ALK5, together with altered Smad signaling, has been associated with chondrocyte dysfunction during OA progression. This study examined whether eugenol attenuates IL-1β-induced ECM dysregulation in primary human chondrocytes and whether ALK1/ALK5-associated Smad signaling contributes to this response. Primary human chondrocytes were treated to IL-1β in the presence or absence of eugenol and analyzed using RT-qPCR, Western blotting, immunofluorescence, exploratory RNA sequencing, and siRNA-mediated loss-of-function experiments. Receptor-expression profiles were also assessed in juvenile and aged mouse cartilage and in cartilage from a previously established ACLT cohort. Eugenol attenuated IL-1β-induced decreases in ACAN, COL2A1, and SOX9 expression and reduced IL-1β-induced increases in MMP13, ADAMTS5, COL10A1, RUNX2, and IL-6 immunoreactivity. At the protein level, eugenol increased COL2A1 abundance and reduced MMP13 and ADAMTS5 level. Exploratory pairwise RNA-seq analysis identified IL-1β-responsive genes that were directionally counter-regulated by both eugenol concentrations; these genes were enriched in inflammatory, cell-behavior, and TGF-β/BMP-related pathways. Prolonged IL-1β exposure increased the ALK1/ALK5 protein ratio, primarily because ALK5 declined more markedly than ALK1. Eugenol treatment was associated with a lower ALK1/ALK5 ratio, reduced Smad1/5/9 phosphorylation, and increased Smad2/3 phosphorylation. ALK1 knockdown phenocopied selected eugenol-associated ECM responses, whereas ALK5 knockdown weakened several matrix-restorative and anti-catabolic responses. Computational docking, short molecular-dynamics simulations, and Y279A/H280A mutagenesis provided exploratory observations but did not demonstrate direct eugenol–ALK1 binding. In mouse cartilage, receptor-expression profiles differed between juvenile and aged animals, and eugenol partially normalized ACLT-associated changes in ALK1 and ALK5 immunostaining. These findings indicate that eugenol attenuates IL-1β-induced ECM dysregulation in chondrocytes. Modulation of ALK1/ALK5-associated Smad signaling may contribute to this response; however, direct target engagement and ligand-specific signaling mechanisms remain to be established.

## 1 Introduction

Osteoarthritis (OA) is the most prevalent degenerative joint disease and a major cause of chronic pain, impaired mobility, and disability worldwide (Hunter and Bierma-Zeinstra, 2019; Katz et al., 2021; GBD, 2021 Osteoarthritis Collaborators, 2023). Although OA is now recognized as a whole-joint disease involving articular cartilage, subchondral bone, synovium, meniscus, and periarticular tissues, progressive degeneration of articular cartilage remains a central pathological feature (Loeser et al., 2012; Chen et al., 2017; Yao et al., 2023). The structural integrity and biomechanical function of cartilage depend on ECM homeostasis, which is maintained primarily by chondrocytes. Disruption of chondrocyte-mediated ECM homeostasis is therefore a key event in OA initiation and progression.

Under inflammatory, mechanical, and aging-associated stress, chondrocytes shift from a matrix-preserving phenotype toward a catabolic, hypertrophic-like, and inflammatory state. This transition is characterized by reduced expression of major cartilage matrix components, including aggrecan and type II collagen, and increased production of ECM-degrading enzymes, such as matrix metalloproteinase 13 (MMP13) and a disintegrin and metalloproteinase with thrombospondin motifs 5 (ADAMTS5) (Chen et al., 2017; Yao et al., 2023; Tan et al., 2025). Type II collagen, encoded by *COL2A1*, is the predominant collagenous component of hyaline cartilage and is widely used as a core readout of cartilage matrix integrity. MMP13 is particularly relevant because it directly mediates type II collagen degradation and has been closely linked to OA cartilage destruction (Blaney Davidson et al., 2009; Little and Fosang, 2010). In addition, SOX9 supports chondrocytic phenotype maintenance and matrix gene expression, whereas COL10A1 and RUNX2 are commonly associated with hypertrophic-like and degenerative chondrocyte remodeling. Thus, simultaneous assessment of anabolic ECM markers, catabolic enzymes, chondrocytic transcriptional markers, and hypertrophy-associated markers provides a more comprehensive evaluation of chondrocyte ECM homeostasis.

Inflammatory cytokines play important roles in promoting cartilage catabolism during OA. Among them, IL-1β is widely used to establish an inflammatory-catabolic chondrocyte model because it induces ECM degradation, suppresses matrix synthesis, and activates inflammatory mediators (Woodell-May and Sommerfeld, 2020; Sanchez-Lopez et al., 2022; Yao et al., 2023). Although current clinical management of OA mainly focuses on pain relief and functional improvement, effective disease-modifying therapies capable of preserving cartilage structure and restoring matrix homeostasis remain limited (Latourte et al., 2020; Katz et al., 2021; Tang et al., 2025). Therefore, identifying compounds and signaling mechanisms that counteract inflammatory-catabolic chondrocyte activation remains an important research priority.

TGF-β signaling plays a context-dependent role in cartilage development, maintenance, and degeneration (van der Kraan, 2017; Thielen et al., 2019; Yao et al., 2023). In healthy adult cartilage, TGF-β predominantly signals through ALK5, leading to Smad2/3 activation, which supports chondrocyte homeostasis, suppresses terminal differentiation, and promotes matrix synthesis (Finnson et al., 2010; Blaney Davidson et al., 2015; van der Kraan, 2017; Thielen et al., 2019). In contrast, during aging and OA progression, TGF-β/BMP-related signaling becomes dysregulated, with a relative shift toward ALK1-associated Smad1/5/9 activation, historically referred to as Smad1/5/8 signaling. This signaling profile has been associated with chondrocyte hypertrophy, matrix catabolism, and cartilage degeneration (Blaney Davidson et al., 2009; Finnson et al., 2010; van der Kraan, 2017; Thielen et al., 2023). Importantly, an increased ALK1/ALK5 ratio has been reported to contribute to elevated MMP13 expression in human and mouse OA cartilage, directly linking receptor-level signaling imbalance to cartilage matrix catabolism (Blaney Davidson et al., 2009). Thus, the balance between ALK1-associated Smad1/5/9 signaling and ALK5-associated Smad2/3 signaling may be an important determinant of chondrocyte phenotype and ECM homeostasis.

Natural bioactive compounds have attracted attention as potential modulators of inflammatory and catabolic signaling in OA (Sheng et al., 2025; Ma et al., 2025). Eugenol is a naturally occurring phenolic compound with reported anti-inflammatory, antioxidant, and cytoprotective properties (Barboza et al., 2018; Ulanowska and Olas, 2021). Previous studies, including our own, have suggested that eugenol may attenuate chondrocyte injury and OA-like pathological changes in experimental models. In a previous ACLT-based eugenol study, we reported that eugenol improved cartilage histological outcomes and modulated ECM-related markers *in vivo* (Wu et al., 2023). However, whether eugenol modulates receptor-level TGF-β/BMP-related signaling, particularly the OA-associated ALK1/ALK5 imbalance, remains unclear.

In the present study, we investigated whether eugenol attenuates IL-1β-induced ECM dysregulation in primary human chondrocytes and whether ALK1/ALK5-associated Smad signaling contributes to this response. We assessed ECM anabolic markers, catabolic enzymes, chondrocytic and hypertrophic markers, inflammatory activation, transcriptomic changes, ALK1/ALK5 expression, and downstream Smad phosphorylation. ALK1 and ALK5 loss-of-function experiments were performed to evaluate the functional contribution of this receptor-associated signaling axis. In addition, receptor-level ALK1/ALK5 changes were examined in mouse cartilage, including cartilage from juvenile and aged mice and mice with ACLT-induced OA. Computational docking, molecular dynamics simulation, and mutagenesis were used only as exploratory approaches to generate hypotheses regarding a putative ALK1 region associated with eugenol responsiveness, and were not interpreted as evidence of direct eugenol–ALK1 binding.

## 2 Materials and methods

### 2.1 Primary human chondrocyte culture and establishment of an OA-like inflammatory model

Primary human chondrocytes were purchased from Procell Life Science and Technology Co., Ltd. (Wuhan, China) and cultured in Human Chondrocyte Medium (Procell) at 37 °C in a humidified incubator containing 5% CO_2_. Cells were passaged with trypsin-EDTA (Gibco, Grand Island, NY, United States) when they reached 80%–90% confluence. To minimize phenotypic drift and dedifferentiation, early-passage cells (passages 1–5) were used for all experiments, and passage numbers were kept comparable among experimental groups.

To establish an OA-like inflammatory model, chondrocytes were cultured under low-serum conditions in DMEM/F-12 medium (Gibco, Grand Island, NY, United States) containing 1% fetal bovine serum (FBS). Cells were pretreated with eugenol (TargetMol, Shanghai, China) at 0, 25, 50, or 100 μg/mL for 2 h, followed by stimulation with recombinant human IL-1β (Novoprotein, Suzhou, China) at 10 ng/mL. Unless otherwise specified, cells were harvested 48 h after IL-1β stimulation. Vehicle-matched controls were included in each experiment, and the final vehicle concentration was kept identical among groups.

The main working concentration of eugenol, 50 μg/mL, was selected based on our previously published dose-response and CCK-8 cytotoxicity data, which showed that 48-h exposure to 50 μg/mL eugenol did not significantly reduce chondrocyte viability under comparable culture conditions (Wu et al., 2023). No additional viability assay was performed under the exact treatment conditions used in the present study.

### 2.2 Reverse transcription quantitative PCR (RT-qPCR) analysis

Primary human chondrocytes were seeded in 6-well plates at a density of 1.5 × 10^5^ to 2.0 × 10^5^ cells per well. After the indicated treatments, total RNA was extracted using the MiniBEST Universal RNA Extraction Kit (TaKaRa, Kusatsu, Japan) according to the manufacturer’s instructions. RNA concentration and purity were measured using a NanoDrop Eight spectrophotometer (Thermo Fisher Scientific, Waltham, MA, United States). Complementary DNA (cDNA) was synthesized using the PrimeScript™ RT reagent Kit with gDNA Eraser (TaKaRa, Kusatsu, Japan) to remove genomic DNA contamination.

RT-qPCR was performed using TB Green® Premix Ex Taq™ (Tli RNaseH Plus) (TaKaRa, Kusatsu, Japan) on an ABI QuantStudio 5 Real-Time PCR System (Applied Biosystems, Foster City, United States). Each reaction was performed with at least three technical replicates. Relative mRNA expression levels were calculated using the 2^−ΔΔCt^ method, with GAPDH used as the internal reference gene, as specified for each experiment.

To evaluate ECM metabolism and chondrocyte phenotypic changes, RT-qPCR was performed for *ACAN* and *COL2A1* as anabolic ECM markers, *MMP13* and *ADAMTS5* as catabolic ECM markers, *SOX9* as a chondrocytic phenotype-associated transcription factor, and *COL10A1* and *RUNX2* as hypertrophy-associated markers. Primer sequences are listed in Table 1.

**TABLE 1:** Primer sequences used for RT-qPCR.

Target gene	Primer name	Sequence (5'- 3′)
*ACAN*	ACAN-F	ACT​CTG​GGT​TTT​CGT​GAC​TCT
*ACAN*	ACAN-R	ACA​CTC​AGC​GAG​TTG​TCA​TGG
*COL2A1*	COL2A1-F	TGG​ACG​ATC​AGG​CGA​AAC​C
*COL2A1*	COL2A1-R	GCT​GCG​GAT​GCT​CTC​AAT​CT
*MMP13*	MMP13-F	ACT​GAG​AGG​CTC​CGA​GAA​ATG
*MMP13*	MMP13-R	GAA​CCC​CGC​ATC​TTG​GCT​T
*ADAMTS5*	ADAMTS5-F	GAA​CAT​CGA​CCA​ACT​CTA​CTC​CG
*ADAMTS5*	ADAMTS5-R	CAA​TGC​CCA​CCG​AAC​CAT​CT
*SOX9*	SOX9-F	AGC​GAA​CGC​ACA​TCA​AGA​C
*SOX9*	SOX9-R	CTG​TAG​GCG​ATC​TGT​TGG​GG
*COL10A1*	COL10A1-F	ATG​CTG​CCA​CAA​ATA​CCC​TTT
*COL10A1*	COL10A1-R	GGT​AGT​GGG​CCT​TTT​ATG​CCT
*RUNX2*	RUNX2-F	CCG​CCT​CAG​TGA​TTT​AGG​GC
*RUNX2*	RUNX2-R	GGG​TCT​GTA​ATC​TGA​CTC​TGT​CC
*ALK1*	ALK1-F	CGA​GGG​ATG​AAC​AGT​CCT​GG
*ALK1*	ALK1-R	GTC​ATG​TCT​GAG​GCG​ATG​AAG
*ALK5*	ALK5-F	ACG​GCG​TTA​CAG​TGT​TTC​TG
*ALK5*	ALK5-R	GCA​CAT​ACA​AAC​GGC​CTA​TCT​C
*GAPDH*	GAPDH-F	GGA​GCG​AGA​TCC​CTC​CAA​AAT
*GAPDH*	GAPDH-R	GGC​TGT​TGT​CAT​ACT​TCT​CAT​GG

### 2.3 Western blotting

Primary human chondrocytes were seeded in 6-well plates at a density of 1.5 × 10^5^ to 2.0 × 10^5^ cells per well. After the indicated treatments, total protein was extracted using a Total Protein Extraction Kit (KeyGen Biotech, Nanjing, China). Cell lysates were centrifuged at 12,000 rpm for 10 min at 4 °C, and the supernatants were collected. Protein concentrations were determined using a BCA Protein Assay Kit (Solarbio, Beijing, China) on a BioTek Cytation 5 multi-mode reader (BioTek Instruments, Winooski, VT, United States).

Protein samples were mixed with NuPAGE™ LDS Sample Buffer (4X) (Invitrogen, Carlsbad, CA, United States), boiled for 5 min, and separated on NuPAGE™ 4%–12% Bis-Tris Mini Protein Gels (Invitrogen, Carlsbad, United States). Equal amounts of protein (20 μg per lane) were loaded and transferred onto polyvinylidene fluoride (PVDF) membranes. Membranes were blocked with 5% non-fat dried milk for total proteins or with 5% BSA for phosphoprotein detection at room temperature for 1 h and then incubated overnight at 4 °C with primary antibodies.

For ECM-related protein analysis, Western blotting was performed for COL2A1, MMP13, and ADAMTS5 using lysates from the Control, IL-1β, and IL-1β + eugenol groups. The following primary antibodies were used: GAPDH (ab9485, Abcam, Cambridge, United Kingdom), β-actin (GB15003, Servicebio, Wuhan, China), COL2A1 (ab188570, Abcam, Cambridge, United Kingdom), MMP13 (ab39012, Abcam, Cambridge, United Kingdom), ADAMTS5 (ab41037, Abcam, Cambridge, United Kingdom), ALK1 (14745-1-AP, Proteintech, Wuhan, China), ALK5 (84453-1-RR, Proteintech, Wuhan, China), phospho-Smad1/5/9 (#13820, Cell Signaling Technology, Danvers, MA, United States), Smad1 (#6944, Cell Signaling Technology, Danvers, MA, United States), phospho-Smad2/3 (#8828, Cell Signaling Technology, Danvers, MA, United States), and Smad2/3 (#8685, Cell Signaling Technology, Danvers, MA, United States). Above all primary antibodies were diluted at 1:1000.

After washing with TBST, membranes were incubated with the appropriate horseradish peroxidase (HRP)-conjugated secondary antibody (1:20,000; ab6721, Abcam, Cambridge, United Kingdom) for 1 h at room temperature. Protein bands were visualized using SuperSignal™ West Pico PLUS Chemiluminescent Substrate (Thermo Fisher Scientific, Waltham, MA, United States) and captured using a Tanon imaging system (Tanon Science and Technology, Shanghai, China). Band intensities were quantified using ImageJ software. For ALK1, ALK5, COL2A1, MMP13, and ADAMTS5 were normalized to GAPDH or β-actin. For Smad signaling analysis, phosphorylated Smad1/5/9 and phosphorylated Smad2/3 were normalized to their corresponding total Smad proteins. Quantitative results were obtained from three independent biological replicates.

For tissue-level Western blotting, articular cartilage was carefully dissected, homogenized in protein extraction buffer, and processed using the same Western blotting procedure described above.

### 2.4 Immunofluorescence (IF) staining

Primary human chondrocytes were seeded in 12-well plates at a density of 8 × 10^4^ cells per well. After the indicated treatments, cells were washed with phosphate-buffered saline (PBS) and fixed with 4% paraformaldehyde (Solarbio, Beijing, China) for 15 min at room temperature. Cells were then permeabilized with 0.3% Triton X-100 (Sigma-Aldrich, St. Louis, MO, United States) for 10 min and blocked with 5% bovine serum albumin (BSA; Solarbio, Beijing, China) in PBS for 1 h at room temperature. Cells were incubated overnight at 4 °C with primary antibodies against MMP13 (18165-1-AP, Proteintech, Wuhan, China), IL-6 (21865-1-AP, Proteintech, Wuhan, China), ADAMTS5 (ab41037, Abcam, Cambridge, United Kingdom), and ACAN (13880-1-AP, Proteintech, Wuhan, China), all diluted 1:200 in blocking buffer. After washing, cells were incubated with appropriate fluorophore-conjugated secondary antibody (ab150077, Abcam, Cambridge, United Kingdom) for 1 h at room temperature in the dark. Nuclei were counterstained with DAPI at 1 μg/mL for 10 min.

Fluorescence images were acquired using an ImageXpress Micro high-content imaging system (Molecular Devices, San Jose, CA, United States). For each marker, images were acquired using identical exposure settings across all groups. Mean fluorescence intensity and the percentage of positively stained cells were quantified using ImageJ software.

For *in vitro* IF quantification, three independent cell-culture experiments were performed. In each independent experiment, at least three randomly selected fields per group were acquired. Field-level measurements were averaged to generate one value for each independent biological replicate. Statistical analysis was performed using replicate-level values rather than treating individual microscopic fields as independent biological replicates.

### 2.5 Animals and ACLT-induced OA model

All animal procedures used to generate the samples analyzed in this study were performed in accordance with institutional guidelines for the care and use of laboratory animals and were approved by the Institutional Animal Care and Use Committee of Beijing Jishuitan Hospital, Capital Medical University, Beijing, China, under approval number 202103-02.

For basal comparison of receptor expression in articular cartilage, male C57BL/6 mice aged 2 weeks or 18 months were used, with n = 6 mice per group. Because the articular cartilage of 2-week-old mice is developmentally immature, this comparison was interpreted as an age-associated receptor-profile comparison rather than as a direct comparison between mature adult and aged cartilage.

For the analysis of ACLT-induced OA, knee joint samples were obtained from a previously established ACLT mouse cohort reported by Wu et al. (2023). Therefore, the ACLT-related animal modeling and treatment procedures were not repeated in the present study. In the original animal experiment, 8-week-old male C57BL/6N mice were randomly assigned to the following groups: normal, sham, ACLT + vehicle, ACLT + low-dose eugenol, and ACLT + high-dose eugenol, with n = 5 mice per group. No formal *a priori* power calculation was performed. The ACLT model was established as previously described, with minor modifications, based on established post-traumatic osteoarthritis protocols (Cai et al., 2020; Wang et al., 2020; Wu et al., 2023). Briefly, mice were anesthetized by intramuscular injection of dexmedetomidine (0.25 mg/kg) and ketamine (25 mg/kg). Adequate depth of anesthesia was confirmed by the absence of pedal withdrawal reflex before surgery. The left knee joint was shaved and sterilized. In the ACLT groups, the left knee joint was exposed through a lateral parapatellar incision, and the anterior cruciate ligament was transected under direct visualization. In the sham group, the joint was exposed and sutured without ligament transection. The normal group received no surgical intervention. Penicillin was administered intraperitoneally during the first 3 days after surgery to reduce the risk of infection. Body weight was monitored weekly throughout the study period, and baseline and endpoint body weights were summarized for group-level comparison. Eugenol was dissolved in a vehicle containing 10% DMSO, 30% PEG 300, 5% Tween-80, and 55% saline. Beginning 2 weeks after ACLT surgery, mice in the eugenol-treated groups received intraperitoneal injections of eugenol at 5 or 10 mg/kg in a total volume of 100 μL once weekly for 6 consecutive weeks, from postoperative week 2 to week 8. Mice in the sham and ACLT + vehicle groups received an equal volume of vehicle according to the same schedule. The doses of 5 and 10 mg/kg were defined as low- and high-dose regimens, respectively, and were selected based on previous rodent studies showing pharmacological efficacy and acceptable tolerability of eugenol at comparable dose ranges (Guénette et al., 2007; Magalhães et al., 2010; Yogalakshmi et al., 2010). The once-weekly administration schedule was selected with reference to the pharmacokinetic data reported by Guénette et al. (2007), which demonstrated rapid *in vivo* metabolism and clearance of eugenol after systemic administration. Accordingly, this regimen was used as a repeated intermittent-exposure protocol during the 6-week postoperative OA-progression period rather than as a strategy to maintain continuous steady-state eugenol concentrations. Therefore, this regimen was not intended to maintain continuous steady-state plasma, synovial fluid, or cartilage concentrations of eugenol. Instead, it was implemented as a repeated intermittent exposure protocol during the 6-week postoperative OA progression period. This schedule was also intended to minimize repeated handling- and injection-related stress associated with more frequent intraperitoneal administration. Predefined exclusion criteria included severe postoperative infection, unexpected death before the planned endpoint, marked body-weight loss, surgical failure, or technical failure during tissue fixation, decalcification, embedding, sectioning, or staining. No animals met the predefined exclusion criteria, and all animals assigned to each group were included in the final analysis. At the experimental endpoint, mice were deeply anesthetized by intramuscular injection of dexmedetomidine hydrochloride and ketamine hydrochloride at final doses of 0.3 mg/kg and 30 mg/kg, respectively. Deep anesthesia was confirmed by the absence of pedal withdrawal and corneal reflexes, followed by cervical dislocation. Death was confirmed by cessation of respiration and heartbeat before knee joints were collected for subsequent analyses.

### 2.6 Tissue processing, immunohistochemistry, image quantification, and animal-study reporting

Animal experiments were reported in accordance with the ARRIVE guidelines. Randomization was performed using a random-number method before surgery and treatment allocation. Investigators responsible for image acquisition, immunohistochemical quantification, and data analysis were blinded to group allocation. Slides and images were coded before quantification, and group information was decoded only after completion of analysis.

At the experimental endpoint, knee joints were harvested and fixed in 4% paraformaldehyde for 48 h, followed by decalcification in 30% formic acid at room temperature for 24–72 h. Specimens were then dehydrated, embedded in paraffin, and sectioned at 5 μm.

For IHC, paraffin sections were deparaffinized, rehydrated, and subjected to antigen retrieval using citrate buffer, pH 6.0, by microwave heating. Endogenous peroxidase activity was blocked with 3% H_2_O_2_ for 10 min at room temperature, followed by blocking with 5% normal goat serum for 30 min. Sections were incubated overnight at 4 °C with primary antibodies against ALK1 and ALK5. The catalog numbers, manufacturers, and working dilutions of these antibodies are listed in Table 2. After washing, sections were incubated with appropriate HRP-conjugated secondary antibodies and visualized using DAB substrate. Nuclei were counterstained with hematoxylin.

**TABLE 2:** Primary antibodies used for immunohistochemistry (IHC).

Target antibody	Host species	Manufacturer	Catalog number	Working dilution
ALK1	Rabbit	Proteintech	14745-1-AP	1:150
ALK5	Rabbit	Proteintech	84453-1-RR	1:200

Positive staining was quantified as the percentage of positively stained cells using ImageJ software. For tissue-level IHC quantification, the animal was used as the statistical unit rather than the microscopic field. For each mouse, three sections were analyzed, and three randomly selected fields were quantified per section. Field-level values were first averaged within each section, and section-level values were then averaged to generate one value per mouse. This mouse-level value was used as one biological replicate for statistical analysis.

In the present study, these samples were used for ALK1/ALK5 receptor-level assessment by immunohistochemistry and Western blotting. Structural histological outcomes, including H&E staining, Safranin O/Fast Green staining, proteoglycan-loss assessment, and OARSI scoring, were not newly generated in the present study and have been reported previously by Wu et al. (2023).

### 2.7 Computational prediction of eugenol-associated candidate proteins using SuperPred

Putative eugenol-associated protein candidates were predicted using the SuperPred webserver (https://prediction.charite.de/superpred/). Structural information for eugenol was obtained from the PubChem database (https://pubchem.ncbi.nlm.nih.gov/). Eugenol has a PubChem CID of 3314, and its canonical SMILES is COC1=C(C=CC(=C1)CC=C)O. The SMILES string was submitted to the Target Prediction module of SuperPred using the default parameters. Predicted targets were collected with their target names, UniProt IDs, prediction probabilities, and model accuracies. Proteins with a prediction probability ≥50% and a model accuracy ≥80% were considered computational candidate proteins. These proteins were regarded as hypothesis-generating candidates rather than experimentally validated direct targets of eugenol. Candidate targets were standardized using UniProt to confirm official protein names, gene symbols, and aliases. Selected candidates were subsequently used for molecular docking and molecular dynamics simulation.

### 2.8 Acquisition and preparation of candidate target structures

Based on SuperPred target prediction and published evidence implicating TGF-β/BMP signaling in OA pathophysiology, ACVRL1/ALK1 was selected as a candidate receptor for molecular docking analysis. This *in silico* selection was used to prioritize a mechanistically relevant receptor for hypothesis-generating analysis and was not considered evidence of direct binding between eugenol and ALK1.

The three-dimensional structure of ACVRL1/ALK1 was obtained from the AlphaFold Protein Structure Database (https://alphafold.ebi.ac.uk/) using UniProt ID P37023 and model ID AF-P37023-F1. The reliability of the predicted structure was evaluated according to the per-residue confidence score, pLDDT, provided by AlphaFold. The full-length ACVRL1/ALK1 model was used for subsequent blind docking analysis.

The protein structure was preprocessed by removing unnecessary atoms, adding hydrogen atoms, assigning charges, and performing energy minimization. The three-dimensional structure of eugenol was downloaded from PubChem (https://pubchem.ncbi.nlm.nih.gov/); hydrogen atoms were added, and energy minimization was performed before docking.

### 2.9 Molecular docking and molecular dynamics simulation

Molecular docking was performed using AutoDock Vina version 1.2.2. Whole-protein blind docking was conducted between eugenol and ACVRL1/ALK1. The docking grid box was set to cover the entire ALK1 structure, allowing the search for putative ligand-compatible regions without predefining a specific binding pocket. The optimal docking pose was selected based on predicted binding affinity and conformational plausibility. Protein–ligand interactions were visualized using PyMOL and Discovery Studio Visualizer. All docking-derived interactions were considered computational predictions and were not interpreted as evidence of experimentally confirmed ligand–protein binding or cellular target engagement.

Molecular dynamics simulations were performed using GROMACS. The ALK1 protein was parameterized using the CHARMM36m force field, and the topology parameters of eugenol were generated using the CGenFF server. The docked eugenol-ALK1 complex was solvated in a cubic box using the TIP3P water model, and counterions were added to neutralize the system. The system was then subjected to energy minimization, NVT equilibration, NPT equilibration, and subsequent molecular dynamics simulation. Root mean square deviation (RMSD) trajectories were generated to evaluate the dynamic behavior of the predicted eugenol-ALK1 complex. The 100-ps trajectory was used to examine early structural relaxation, whereas the 10-ns trajectory was used to assess short-timescale dynamic stability. These computational analyses were interpreted as hypothesis-generating evidence only and did not establish direct ligand–protein binding or cellular target engagement between eugenol and ALK1.

### 2.10 RNA sequencing, differential expression analysis, eugenol-reversal analysis, and exploratory Mfuzz clustering

For transcriptomic analysis, primary human chondrocytes were assigned to four groups: Control, IL-1β, IL-1β + 50 μg/mL eugenol, and IL-1β + 100 μg/mL eugenol, with three independent biological replicates per group. Cells in the eugenol-treated groups were pretreated with 50 or 100 μg/mL eugenol for 2 h before IL-1β stimulation and were then cultured under the corresponding treatment conditions for 24 h. IL-1β stimulation was performed as described above. Total RNA was extracted using the MiniBEST Universal RNA Extraction Kit (TaKaRa, Kusatsu, Japan) according to the manufacturer’s instructions. RNA quantity and purity were assessed using a NanoDrop spectrophotometer, and RNA integrity was evaluated using an Agilent 2100 Bioanalyzer (Agilent Technologies, Santa Clara, CA, United States). Only samples with an RNA integrity number (RIN) ≥ 7.0 were used for library preparation. Sequencing libraries were prepared using the NEBNext Ultra II RNA Library Prep Kit for Illumina (New England Biolabs, Ipswich, MA, United States) according to the manufacturer’s protocol. RNA library construction and sequencing were performed by Beijing Zhongkangbo Biotechnology Co., Ltd. (Beijing, China). Briefly, poly(A)+ mRNA was enriched using oligo(dT) magnetic beads, fragmented, reverse-transcribed, end-repaired, adaptor-ligated, and PCR-amplified. Libraries were quality-checked using the Agilent 2100 Bioanalyzer and sequenced on an Illumina NovaSeq 6000 platform to generate 150-bp paired-end reads. Raw sequencing reads were processed using fastp version 0.23.2 for adaptor trimming and low-quality read filtering. Clean reads were aligned to the human reference genome GRCh38 using HISAT2 version 2.2.1. Gene-level read counts were generated using featureCounts version 2.0.3 based on the corresponding human gene annotation file. Differential expression analysis was performed using the limma package version 3.62.2 in R after voom normalization. Direct pairwise comparisons were performed for IL-1β versus Control, IL-1β + eugenol 50 μg/mL versus IL-1β, and IL-1β + eugenol 100 μg/mL versus IL-1β. Differentially expressed genes were defined as genes with a nominal P value <0.05 and |log2FC| > 1, corresponding to FC > 2 for upregulated genes and FC < 0.5 for downregulated genes. Benjamini-Hochberg adjusted P values were calculated and reported in the supplementary tables. Because nominal P values rather than FDR-adjusted P values were used as the primary cutoff, the RNA-seq results were interpreted as exploratory and integrated with downstream enrichment, network analysis, and experimental validation.

IL-1β-responsive genes were defined as differentially expressed genes in the IL-1β group compared with the Control group. Eugenol-counter-regulated genes were defined as IL-1β-responsive genes that were significantly altered in the opposite direction in the IL-1β + eugenol group compared with the IL-1β group using the same differential expression threshold. Specifically, genes upregulated by IL-1β were considered reversed if they were significantly downregulated by eugenol treatment, whereas genes downregulated by IL-1β were considered reversed if they were significantly upregulated by eugenol treatment. Reversed genes were identified separately for the 50 and 100 μg/mL eugenol groups. Commonly reversed genes were defined as the intersection of reversed genes shared by both eugenol doses.

The voom-normalized expression matrix of DEGs identified from the direct differential comparisons was used for exploratory clustering analysis with the Mfuzz package in R. For Mfuzz clustering, expression values were averaged within each treatment group to generate a gene-by-condition matrix. Before clustering, the expression matrix was standardized using the standardise function so that each gene had a comparable expression scale across treatment conditions. Fuzzy c-means clustering was then performed. The fuzzification parameter m was estimated using the mestimate function, and the number of clusters c was selected based on the minimum centroid distance, clustering stability, and biological interpretability of the expression patterns.

Mfuzz clustering was used to explore condition-associated gene expression patterns across the Control, IL-1β + eugenol 100 μg/mL, IL-1β + eugenol 50 μg/mL, and IL-1β groups. The group order was used only to visualize expression changes across treatment conditions. Because these positions represented treatment conditions rather than chronological time points, the Mfuzz results were not interpreted as temporal trajectories. After clustering, each gene was assigned a membership score for each cluster. Genes with a membership score ≥0.5 were defined as core genes of the corresponding cluster. The expression patterns of different clusters were visualized using the mfuzz.plot function. Core genes from each cluster were used for subsequent functional enrichment analysis to explore their potential biological roles.

### 2.11 GO, KEGG, and STRING-based network analysis

Gene Ontology (GO) and Kyoto Encyclopedia of Genes and Genomes (KEGG) enrichment analyses were performed using the clusterProfiler package (version 4.6.2) in R. Gene symbols were converted to Entrez gene IDs using the org.Hs.eg.db annotation package where required. The background gene universe was defined as all expressed genes retained after expression filtering in the RNA-seq dataset. GO enrichment analysis was performed for the biological process (BP), cellular component (CC), and molecular function (MF) categories, and KEGG pathway enrichment analysis was performed using human pathway annotations. Enrichment significance was assessed using the Benjamini–Hochberg method, and terms or pathways with an adjusted P value <0.05 were considered significantly enriched. Enrichment analyses were performed for IL-1β-responsive genes, eugenol-counter-regulated genes, commonly reversed genes, and selected Mfuzz cluster core genes where indicated. The top significantly enriched GO terms in the BP, CC, and MF categories and KEGG pathways were visualized using dot plots and gene-count bar plots generated with the enrichplot package in R. In the dot plots, the x-axis represented the GeneRatio, the y-axis represented enriched GO terms or KEGG pathways, point size represented gene count, and color represented the adjusted P value. In the bar plots, the x-axis represented the number of enriched genes and the y-axis represented enriched GO terms or KEGG pathways.

Protein-protein interaction and functional association networks were constructed using the STRING database version 12.0. The organism was restricted to *Homo sapiens*. Since activin receptor-like kinase 1 (ALK1) is encoded by ACVRL1, the official gene symbol ACVRL1 was used for STRING-based network analysis. For the ALK1-centered network, ACVRL1 was submitted as the query gene, and first-shell functional interactors were retrieved to generate an ALK1-centered interaction network. The minimum required interaction score was set at 0.400, corresponding to the default medium-confidence threshold in STRING. Disconnected nodes were hidden from the final network. Active interaction sources included curated databases, experimental evidence, co-expression, text mining, gene neighborhood, gene fusion, and gene co-occurrence. Network edges represented STRING functional associations rather than necessarily direct physical interactions.

To further evaluate the relationship between ALK1 and TGF-β/Smad signaling, a TGF-β pathway-related ALK1 network was constructed. TGF-β pathway-related genes identified in Mfuzz cluster 13 were submitted to STRING together with ACVRL1. These genes were mapped to official human gene symbols before network construction. Key components of the ALK1/ALK5-Smad signaling axis, including ACVRL1/ALK1, TGFBR1/ALK5, TGFBR2, and SMAD family members, were retained and annotated when present in the network. The same STRING parameters were applied, including the *H. sapiens* organism setting and a minimum interaction score of 0.400.

The resulting networks were visualized and exported directly from the STRING web interface. Nodes represented proteins, and edges represented known or predicted functional associations from the STRING database. ACVRL1/ALK1 was used as the query or seed node and was highlighted in the ALK1-centered and TGF-β pathway-related networks.

### 2.12 ALK1 and ALK5 siRNA knockdown

To evaluate the functional involvement of ALK1/ALK5-Smad signaling in eugenol-mediated ECM protection, siRNA-mediated loss-of-function experiments were performed for both ALK1 and ALK5. Primary human chondrocytes were seeded in 6-well plates and transfected at approximately 70%–80% confluence with ALK1-specific siRNA targeting *ACVRL1*, ALK5-specific siRNA targeting *TGFBR1*, or a non-targeting negative-control siRNA using Lipofectamine 3000 reagent (Thermo Fisher Scientific, Waltham, MA, United States) according to the manufacturer’s instructions. The final siRNA concentration was 30 nM. The siRNA sequences are listed in Table 3.

**TABLE 3:** siRNA sequences used for ALK1/ALK5 loss-of-function experiments in primary human chondrocytes.

Target gene	siRNA name	Sequence (5'- 3′)
*ALK5*	siALK5-F	UAU​CUC​GAG​GAA​UUA​AGU​C
*ALK5*	siALK5-R	GGA​UCA​AGA​AGA​CAC​UAC​A
*ALK1*	siALK1-F	CAG​UGU​UGC​UCA​GAC​ACG​A
*ALK1*	siALK1-R	UCG​UGU​CUG​AGC​AAC​ACU​G

The sequence of the negative-control siRNA, was proprietary and was provided by the manufacturer.

At 48 h after transfection, cells were pretreated with eugenol at 50 μg/mL for 2 h and then stimulated with IL-1β at 10 ng/mL for 48 h. Knockdown efficiency of ALK1 and ALK5 was confirmed at the mRNA and protein levels by RT-qPCR and Western blotting. The expression of ECM-related markers, including ACAN, COL2A1, MMP13, and ADAMTS5, was assessed by RT-qPCR and Western blotting.

### 2.13 Plasmid construction, site-directed mutagenesis, and transient transfection

A full-length human ACVRL1/ALK1 expression construct was generated by cloning the coding sequence of human ACVRL1 (NM_000020.3) into the pcDNA3.1(+) vector backbone containing a C-terminal FLAG tag. Site-directed mutagenesis was performed using the Mut Express II Fast Mutagenesis Kit V2 (Vazyme Biotech Co., Ltd., Nanjing, China) according to the manufacturer’s instructions to generate an ALK1 mutant harboring Tyr279Ala and His280Ala substitutions (Y279A/H280A). All plasmids were verified by Sanger sequencing before use.

For transient transfection, primary human chondrocytes were seeded in 6-well plates and transfected at approximately 70%–80% confluence using Lipofectamine 3000 (Thermo Fisher Scientific, Waltham, MA, United States) according to the manufacturer’s instructions. Briefly, 2 μg plasmid DNA per well was diluted in Opti-MEM and mixed with the transfection reagent. The DNA-lipid complexes were added to the cells for 6 h, after which the medium was replaced with complete growth medium. Cells were allowed to recover for 24 h before subsequent treatments. The corresponding empty pcDNA3.1(+)-Flag vector was used as the control. Overexpression efficiency was confirmed by Western blotting before functional analysis.

In mutagenesis experiments, cells were transfected with empty vector, wild-type ALK1, or Y279A/H280A mutant ALK1, followed by pretreatment with eugenol for 2 h and stimulation with IL-1β for 48 h. The Y279A/H280A mutagenesis analysis was designed to evaluate the functional relevance of the predicted ALK1 region in eugenol-associated cellular responses. This assay was not interpreted as direct evidence of physical binding between eugenol and ALK1, because mutation-induced changes in local protein conformation, stability, or signaling competence cannot be fully excluded.

### 2.14 Statistical analysis

All statistical analyses were performed using GraphPad Prism software version 10.1.2 (GraphPad Software, San Diego, CA, United States). Data are presented as the mean ± SD. Normality was assessed using the Shapiro–Wilk test, and homogeneity of variance was evaluated using Levene’s test. For normally distributed data with equal variance, comparisons between two groups were performed using an unpaired two-tailed Student’s t-test, whereas comparisons among multiple groups were performed using one-way ANOVA followed by Tukey’s *post hoc* test. For data that did not meet the assumptions of normality and/or equal variance, the Mann-Whitney U test or Kruskal–Wallis test followed by Dunn’s multiple-comparison test was used as appropriate. For RNA-seq analysis, multiple testing was corrected using the Benjamini-Hochberg false-discovery-rate method. For *in vitro* experiments, n represents independent biological replicates or independent experiments. For animal experiments and tissue staining quantification, the animal was used as the statistical unit. Multiple microscopic fields and sections from each animal were quantified and averaged to generate a single value for each animal before statistical analysis. Individual microscopic fields were not treated as independent biological replicates. P < 0.05 was considered statistically significant.

## 3 Results

### 3.1 Eugenol attenuates IL-1β-induced changes in ECM-related, hypertrophy-associated, and inflammatory markers in primary human chondrocytes

**FIGURE 1:**
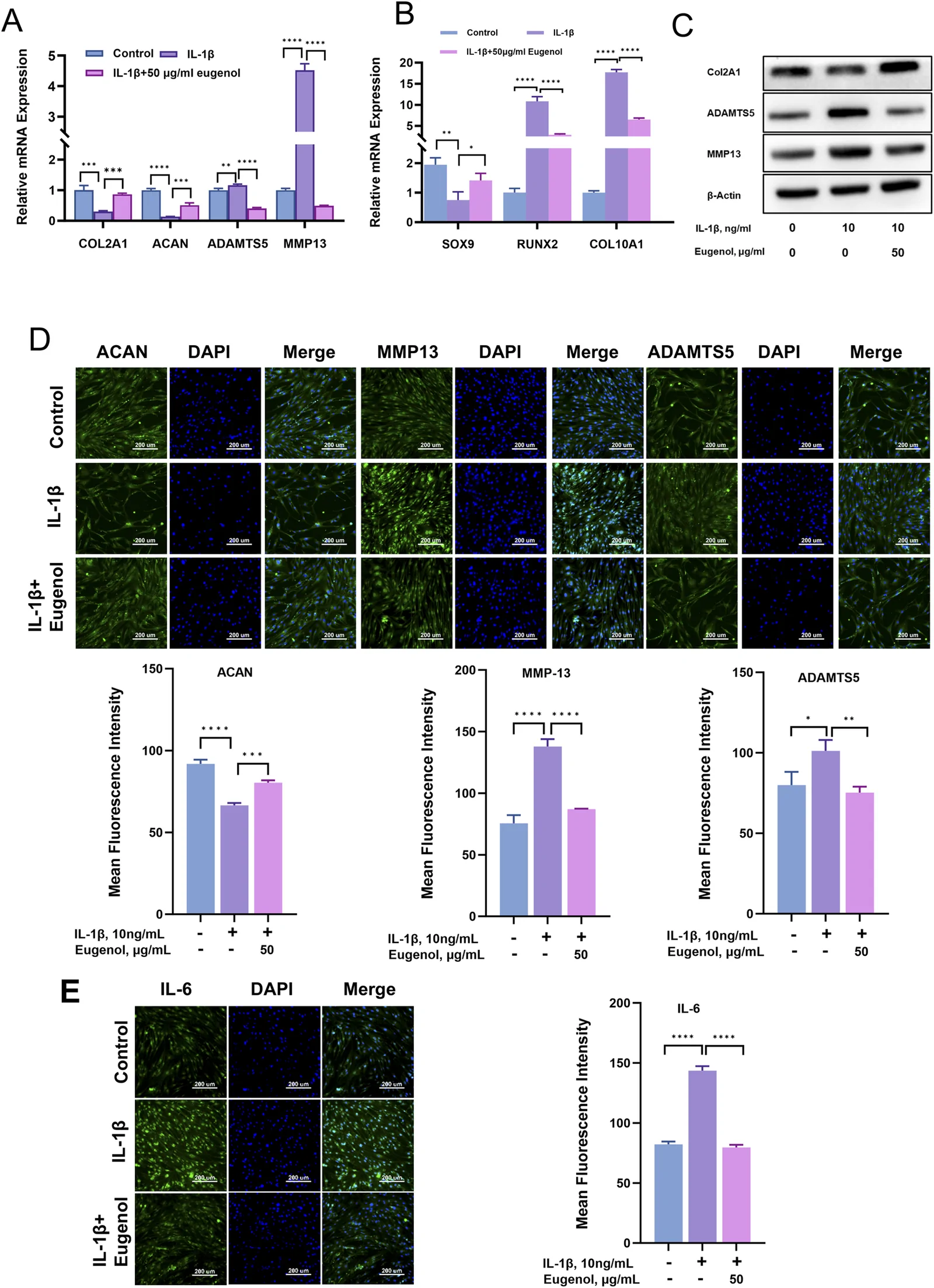
Eugenol preserves ECM homeostasis and attenuates inflammatory activation in IL-1β-stimulated primary human chondrocytes. Primary human chondrocytes were stimulated with 10 ng/mL IL-1β for 48 h in the presence or absence of 50 μg/mL eugenol. **(A)** RT-qPCR analysis of ECM anabolic genes *ACAN* and *COL2A1*, and catabolic genes *MMP13* and *ADAMTS5*. **(B)** RT-qPCR analysis of the chondrocytic marker *SOX9* and hypertrophic markers *COL10A1* and *RUNX2*. **(C)** Western blot analysis and quantification of COL2A1, MMP13, and ADAMTS5 protein levels. **(D)** Representative immunofluorescence images and quantification of mean fluorescence intensity (MFI) for ACAN, MMP13, and ADAMTS5. Scale bar = 200 μm. **(E)** Representative immunofluorescence images and quantification of mean fluorescence intensity (MFI) of IL-6 expression. Scale bar = 200 μm. Data are presented as the mean ± SD from n = 3 independent experiments. Statistical significance was determined by one-way ANOVA followed by Tukey’s *post hoc* test. The main comparisons were Control vs. IL-1β and IL-1β vs. IL-1β + eugenol. Exact pairwise comparisons are indicated by horizontal brackets in the graphs. *P < 0.05, **P < 0.01, ***P < 0.001, and ****P < 0.0001.

Based on our previously published dose-response and cytotoxicity data, 50 μg/mL eugenol was selected as the main working concentration for *in vitro* phenotypic experiments unless otherwise indicated, with vehicle-matched controls included throughout the assays (Wu et al., 2023). Primary human chondrocytes were stimulated with IL-1β at 10 ng/mL for 48 h to establish an OA-like inflammatory-catabolic cell model. To evaluate the effect of eugenol on ECM homeostasis, we first examined representative anabolic matrix markers and catabolic ECM-degrading enzymes at the mRNA level. RT-qPCR analysis showed that IL-1β stimulation significantly upregulated the catabolic genes *MMP13* and *ADAMTS5* and downregulated the matrix-associated genes *ACAN* and *COL2A1*. Eugenol treatment significantly counteracted these IL-1β-induced transcriptional changes, as shown by increased *ACAN* and *COL2A1* mRNA levels and decreased *MMP13* and *ADAMTS5* mRNA levels compared with IL-1β stimulation alone (Figure 1A). These results indicate that eugenol attenuates IL-1β-induced disruption of ECM homeostasis at the transcriptional level.

To further assess whether eugenol preserved the chondrocytic phenotype under inflammatory stress, we examined the expression of the chondrocytic transcription factor *SOX9* and the hypertrophic markers *COL10A1* and *RUNX2*. IL-1β stimulation decreased *SOX9* expression and increased *COL10A1* and *RUNX2* expression. In contrast, eugenol treatment restored *SOX9* expression toward the level observed in control cells and suppressed the IL-1β-induced upregulation of *COL10A1* and *RUNX2* (Figure 1B). These findings suggest that eugenol helps maintain the chondrocytic features and limits hypertrophic-like transcriptional remodeling in IL-1β-stimulated chondrocytes.

We next examined whether these transcriptional changes were reflected at the protein level. Western blot analysis showed that IL-1β reduced the protein abundance of COL2A1 and increased the levels of the catabolic enzymes MMP13 and ADAMTS5. Consistent with the RT-qPCR results, eugenol treatment counteracted these IL-1β-induced alterations, increasing COL2A1 protein levels while decreasing MMP13 and ADAMTS5 protein levels (Figure 1C). These data provide protein-level evidence that eugenol attenuates IL-1β-associated changes in ECM-related anabolic and catabolic markers.

Immunofluorescence staining further supported the ECM-protective effect of eugenol. IL-1β stimulation increased MMP13 and ADAMTS5 immunofluorescence intensity and reduced ACAN immunofluorescence intensity, whereas eugenol attenuated the IL-1β-induced increase in MMP13 and ADAMTS5 and restored ACAN immunofluorescence intensity compared with IL-1β stimulation alone (Figure 1D). In addition, IL-1β markedly increased IL-6 immunofluorescence intensity, while eugenol reduced IL-6 immunofluorescence intensity in IL-1β-stimulated chondrocytes (Figure 1E), indicating that eugenol also alleviated inflammatory activation.

Collectively, these findings indicate that eugenol attenuates IL-1β-induced inflammatory-catabolic changes in primary human chondrocytes by improving ECM-related, hypertrophy-associated, and inflammatory marker profiles.

### 3.2 Exploratory pairwise transcriptomic analysis identifies IL-1β-responsive genes that are directionally counter-regulated by eugenol

Given that the four RNA-seq groups represented distinct treatment conditions rather than consecutive time-series samples, pairwise differential expression analysis was adopted as the primary transcriptomic analysis strategy. IL-1β-responsive genes were first identified by comparing the IL-1β-treated group with the Control group. A total of 2,344 differentially expressed genes (DEGs) were identified in the IL-1β versus Control comparison and defined as IL-1β-responsive genes, including 1,036 upregulated genes and 1,308 downregulated genes following IL-1β stimulation.

To further identify genes counter-regulated by eugenol, the two IL-1β + eugenol co-treatment groups, namely 50 and 100 μg/mL eugenol, were separately compared with the IL-1β group. Genes were defined as directionally counter-regulated by eugenol if they met the DEG criteria in the IL-1β versus Control comparison and showed significant differential expression in the opposite direction in the IL-1β + eugenol versus IL-1β comparison. Specifically, IL-1β-upregulated genes were considered reversed when they were downregulated by eugenol, whereas IL-1β-downregulated genes were considered reversed when they were upregulated by eugenol.

Among the 1,036 IL-1β-upregulated genes, 239 genes were directionally counter-regulated by 50 μg/mL eugenol and 248 genes were reversed by 100 μg/mL eugenol, with 218 genes shared by both doses. Among the 1,308 IL-1βdownregulated genes, 641 genes were directionally counter-regulated by 50 μg/mL eugenol and 725 genes were restored by 100 μg/mL eugenol, with 622 genes shared between the two doses.

**FIGURE 2:**
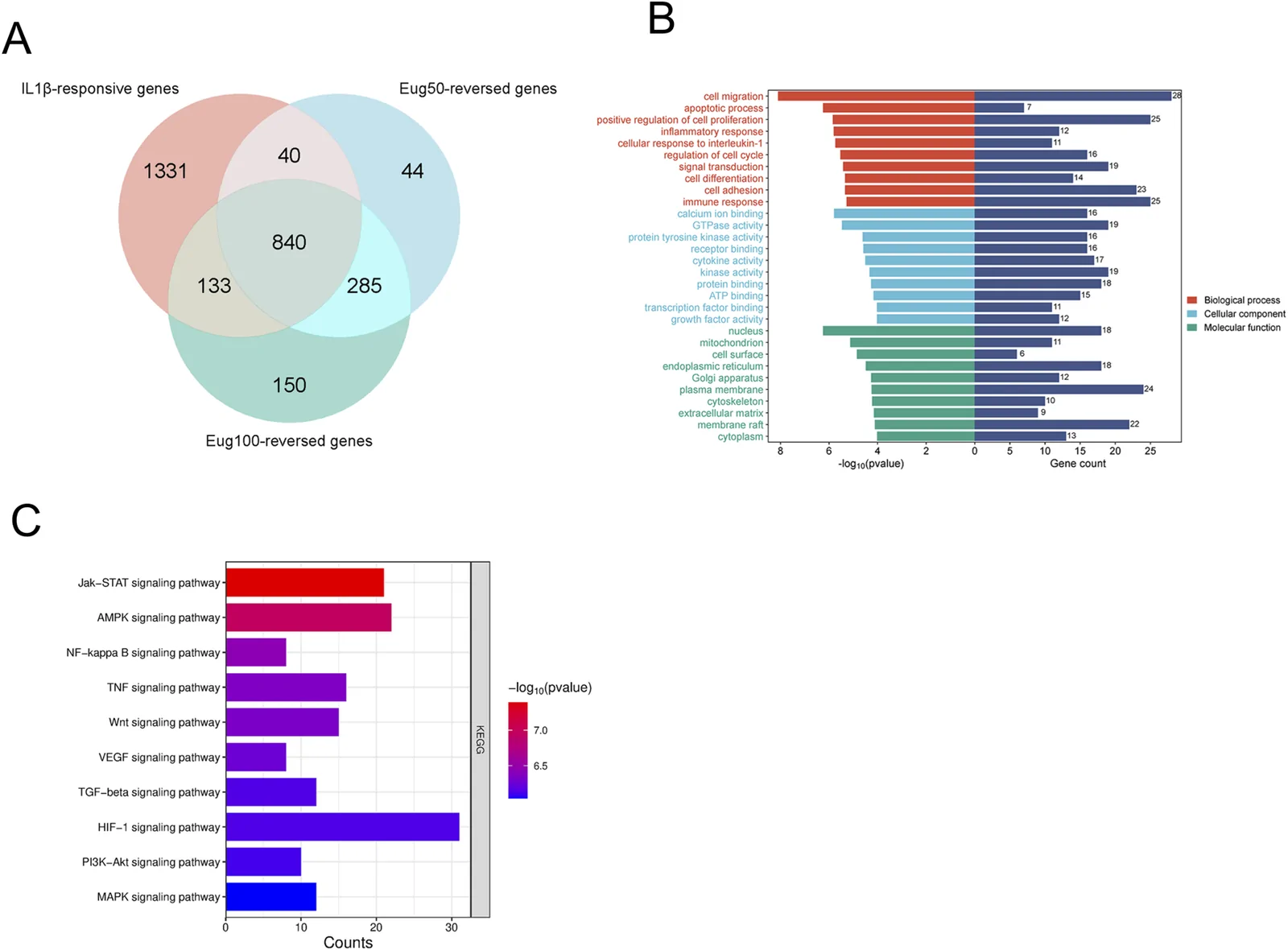
Identification and functional enrichment analysis of eugenol-reversed IL-1β-responsive genes. **(A)** Venn diagram showing the overlap among IL-1β-responsive genes and genes reversed by 50 μg/mL or 100 μg/mL eugenol. IL-1β-responsive genes were identified by comparing the IL-1β group with the Control group. Eugenol-counter-regulated genes were defined as IL-1β-responsive genes that exhibited significant differential expression in the opposite direction in the IL-1β + eugenol group compared with the IL-1β group. A total of 840 genes were commonly reversed by both eugenol doses and were selected as the core gene set for enrichment analysis. **(B)** Gene Ontology (GO) enrichment analysis of the 840 commonly reversed genes, including biological process, molecular function, and cellular component categories. Representative enriched GO terms are shown. **(C)** Kyoto Encyclopedia of Genes and Genomes (KEGG) pathway enrichment analysis of the 840 commonly reversed genes. Enriched pathways included inflammation-, metabolism-, survival-, stress response-, and cell function-related signaling pathways. Bar length represents the number of genes mapped to each pathway, and dot color represents -log10(P value).

Overall, 880 IL-1β-responsive genes were directionally counter-regulated by 50 μg/mL eugenol, whereas 973 genes were directionally counter-regulated by 100 μg/mL eugenol. Among these, 840 genes were commonly reversed by both doses of eugenol, including 218 IL-1β-upregulated genes and 622 IL-1β-downregulated genes. The overlap among IL-1β-responsive genes and genes reversed by the two eugenol doses is shown in Figure 2A. Detailed gene lists are provided in Supplementary Table S2. The 840 commonly reversed genes were selected as the core gene set for subsequent functional enrichment analysis.

### 3.3 Functional enrichment analysis of commonly reversed genes

To further characterize the biological functions and signaling pathways associated with the 840 commonly reversed genes, GO and KEGG pathway enrichment analyses were performed. Representative enriched GO terms and KEGG pathways are shown in Figures 2B,C. GO enrichment analysis indicated that the commonly reversed genes were mainly associated with immune and inflammatory regulation, cell behavior modulation, signal transduction, and cellular structural organization. In the biological process category, the enriched terms included immune response, inflammatory response, cellular response to interleukin-1, cell migration, cell adhesion, positive regulation of cell proliferation, regulation of cell cycle, signal transduction, and cell differentiation. Among these terms, immune response, cell migration, and positive regulation of cell proliferation contained the largest numbers of enriched genes, with 25 genes represented in each of these categories. These findings suggest that eugenol may counter-regulate IL-1β-induced transcriptomic alterations related to inflammatory activation, immune response, and fundamental cellular behaviors.

In the molecular function category, the commonly reversed genes were mainly enriched in binding- and catalytic activity-related functions, including calcium ion binding, receptor binding, cytokine activity, protein tyrosine kinase activity, kinase activity, ATP binding, transcription factor binding, protein binding, and growth factor activity. These results indicate that eugenol may influence IL-1β-induced signaling disturbances by modulating genes involved in receptor-ligand interactions, kinase-dependent signaling, transcriptional regulation, and growth factor-related activity. In the cellular component category, the proteins encoded by commonly counter-regulated genes were broadly distributed across intracellular, membrane-associated, and extracellular compartments, including the Golgi apparatus, endoplasmic reticulum, nucleus, cytoplasm, plasma membrane, membrane raft, cell surface, and extracellular matrix. This broad distribution suggests that the reversed genes may participate in multiple regulatory layers, including intracellular trafficking, membrane-associated signaling, transcriptional regulation, and extracellular matrix remodeling.

Consistent with the GO enrichment results, KEGG pathway enrichment analysis showed that the commonly reversed genes were enriched in multiple inflammation-, metabolism-, survival-, and stress response-related signaling pathways. The major enriched pathways included the JAK-STAT signaling pathway, AMPK signaling pathway, TNF signaling pathway, Wnt signaling pathway, HIF-1 signaling pathway, TGF-β signaling pathway, PI3K-Akt signaling pathway, MAPK signaling pathway, NF-κB signaling pathway, and VEGF signaling pathway. Among these pathways, the HIF-1 signaling pathway contained the largest number of mapped genes, whereas the JAK-STAT and AMPK signaling pathways showed the highest enrichment significance, as reflected by the largest −log10(P value). These pathways are widely involved in inflammatory signaling, metabolic reprogramming, cell proliferation, survival, stress adaptation, and tissue remodeling.

Together, the GO and KEGG enrichment results suggest that eugenol may counter-regulate IL-1β-induced transcriptomic dysregulation through coordinated effects on inflammatory responses, immune-related processes, cell migration and proliferation, metabolic adaptation, and multiple canonical signaling pathways.

### 3.4 Exploratory condition-associated clustering and STRING analysis provide network-level context for ACVRL1/ALK1

To further explore treatment-associated gene expression patterns, Mfuzz fuzzy c-means clustering was performed as an exploratory analysis. This analysis was used to visualize expression-pattern similarities across the four treatment conditions, which were ordered as Control, IL-1β + 100 μg/mL eugenol, IL-1β + 50 μg/mL eugenol, and IL-1β for visualization purposes only. This ordering did not represent chronological progression or a biological time course.

**FIGURE 3:**
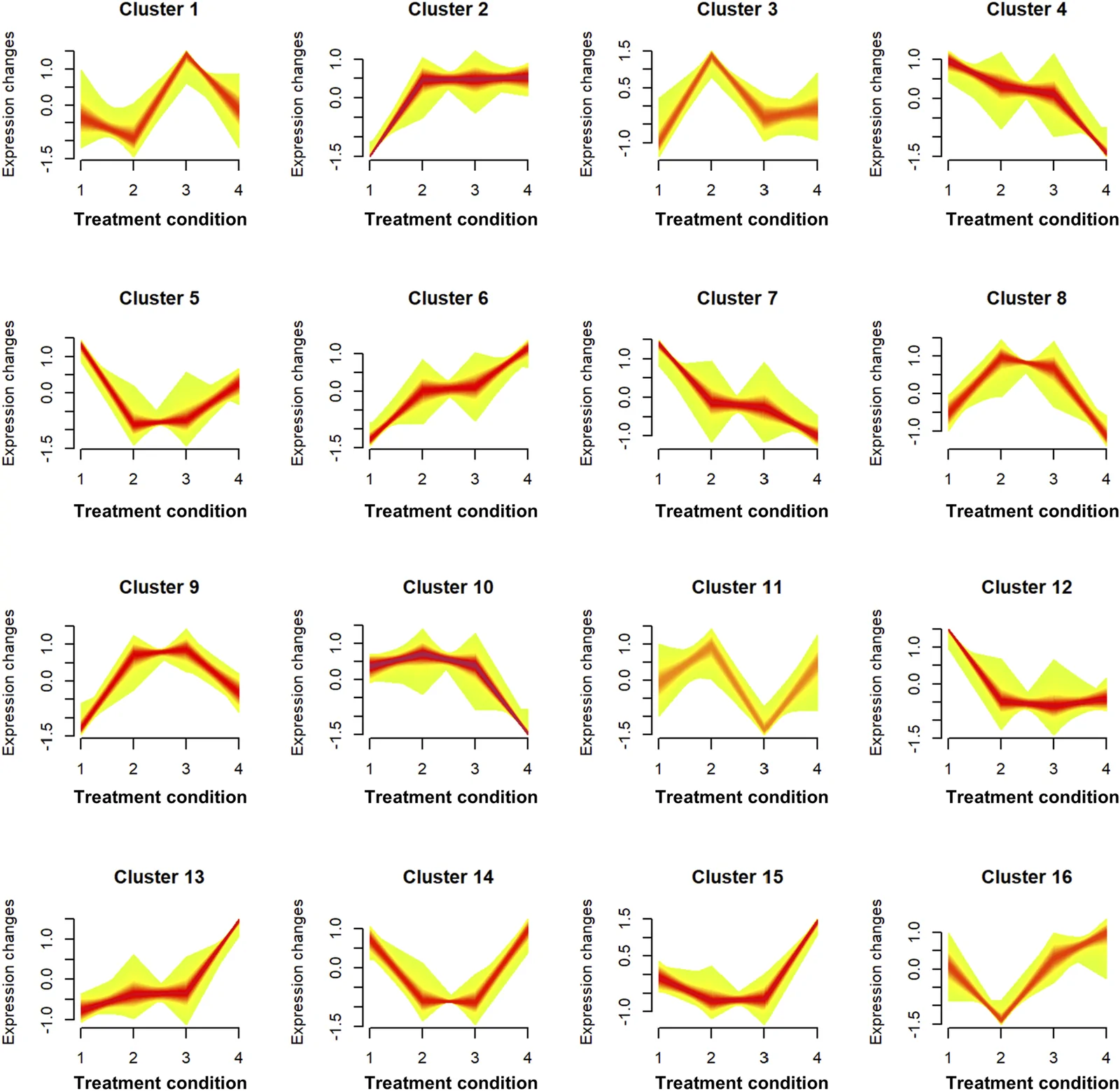
Exploratory Mfuzz clustering of treatment-associated gene expression patterns. Mfuzz fuzzy c-means clustering was performed to visualize treatment-associated expression patterns of differentially expressed genes across the four RNA-seq groups. For visualization only, the groups were ordered as Control, IL-1β + 100 μg/mL eugenol, IL-1β + 50 μg/mL eugenol, and IL-1β. This ordering represents treatment conditions and does not indicate chronological progression or a biological time course. All genes included in the Mfuzz analysis were assigned to 16 clusters, each showing a distinct expression pattern across the four treatment conditions. Thin colored lines represent individual genes, and the bold line represents the cluster centroid. Cluster 13 showed relatively high expression in the IL-1β group and lower expression in the Control and IL-1β + eugenol groups, consistent with an IL-1β-associated transcriptional increase attenuated by eugenol treatment. Mfuzz clustering was used as an exploratory supporting analysis and was not used as the primary basis for downstream target prioritization. On the x-axis, 1–4 correspond to Control, IL-1β + 100 μg/mL eugenol, IL-1β + 50 μg/mL eugenol, and IL-1β, respectively.

All DEGs included in the Mfuzz analysis were assigned to 16 clusters, each showing a distinct expression pattern across the four treatment conditions (Figure 3). Supplementary Table S1 provides the assigned cluster, membership score, number of genes in each cluster, and complete gene lists for all 16 clusters.

Among these clusters, Cluster 13 showed a treatment-associated expression pattern characterized by relatively high expression in the IL-1β-only group and lower expression in the Control and IL-1β + eugenol groups. This pattern was consistent with an IL-1β-associated increase that was attenuated by eugenol treatment. However, because these experimental groups represented discrete treatment conditions rather than a temporal or ordered biological trajectory, and because unsupervised cluster assignment alone is insufficient for rigorous target prioritization, Cluster 13 was considered exploratory supporting information rather than a primary criterion for downstream target selection.

**FIGURE 4:**
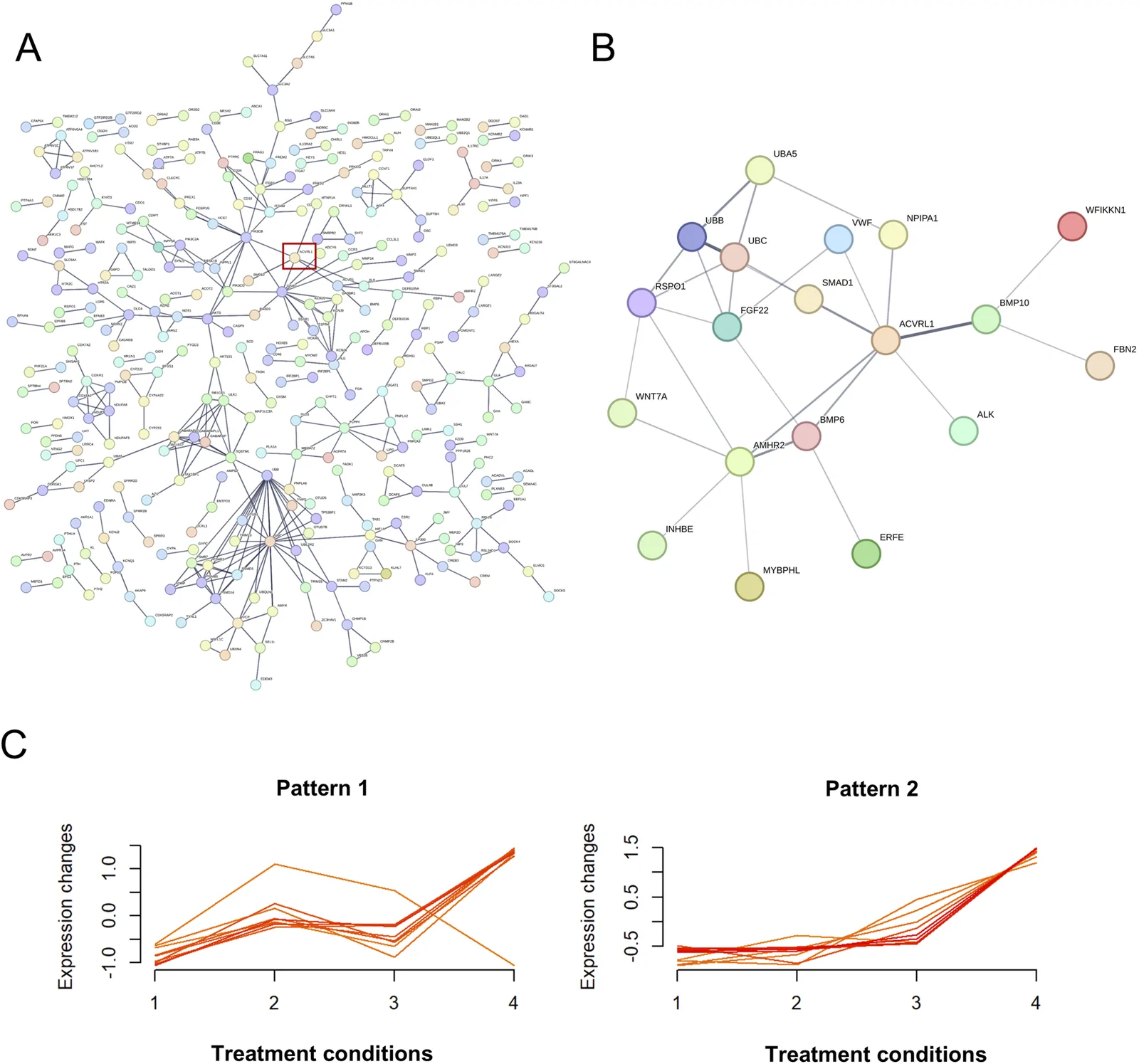
ALK1/ACVRL1-centered protein–protein interaction network analyses. **(A)** ALK1/ACVRL1-centered PPI network generated using the STRING database with ALK1/ACVRL1 as the seed node. **(B)** ALK1/ACVRL1-centered TGF-β/BMP pathway-related PPI network constructed from TGF-β/BMP-Smad-related genes extracted from Mfuzz Cluster 13. ALK1/ACVRL1 was included as the central node. Nodes represent proteins, and edges represent known or predicted STRING functional associations rather than necessarily direct physical interactions. **(C)** Treatment-pattern visualization of ALK1/ACVRL1-associated genes extracted from the STRING-supported subnetwork across the ordered experimental conditions: Control, IL-1β + 100 μg/mL eugenol, IL-1β + 50 μg/mL eugenol, and IL-1β. The group order was used for visualization only and does not represent chronological progression.

To place ACVRL1/ALK1 within a signaling-network context, an ACVRL1/ALK1-centered protein–protein interaction (PPI) network was constructed using the STRING database, with ACVRL1/ALK1 as the seed node. This ALK1-centered network showed functional associations between ACVRL1/ALK1 and multiple TGF-β/BMP-Smad-related components, supporting the relevance of ALK1 as a receptor-level signaling node in pathways implicated in cartilage homeostasis and OA-related matrix remodeling (Figure 4A).

To further connect ALK1 with the treatment-associated transcriptomic pattern, an ALK1-centered TGF-β/BMP pathway-related PPI network was constructed using TGF-β/BMP-Smad pathway-related genes extracted from Mfuzz Cluster 13. ACVRL1/ALK1 was included as the central node, and the resulting network highlighted functional associations between ALK1 and TGF-β/BMP-Smad signaling components within this Cluster 13-derived gene module (Figure 4B). Because Mfuzz clustering was used as an exploratory analysis across discrete treatment conditions rather than chronological time points, this Cluster 13-derived PPI network was interpreted as supportive network-level evidence.

ACVRL1/ALK1-associated genes extracted from the STRING-supported subnetwork were then visualized across the ordered experimental conditions: Control, IL-1β + 100 μg/mL eugenol, IL-1β + 50 μg/mL eugenol, and IL-1β (Figure 4C). This ordering was used only for treatment-pattern visualization and did not represent a temporal trajectory. Two major expression patterns were observed. Pattern 1 showed a heterogeneous treatment-associated profile, whereas Pattern 2 showed a clearer IL-1β-responsive pattern, with strong upregulation in the IL-1β group and relatively low expression in the Control and eugenol-treated groups. These patterns were consistent with eugenol-associated counter-regulation of IL-1β-induced transcriptional changes within the ACVRL1/ALK1-associated subnetwork.

Together, these network and expression-pattern analyses provided exploratory network-level context supporting the relevance of ACVRL1/ALK1 within TGF-β/BMP-Smad-related signaling. These analyses were not interpreted as evidence that ACVRL1/ALK1 is a direct molecular target of eugenol, but they supported subsequent functional evaluation of ALK1/ALK5-associated Smad signaling in IL-1β-stimulated chondrocytes.

### 3.5 Eugenol is associated with partial correction of the IL-1β-induced ALK1/ALK5 receptor imbalance and altered Smad phosphorylation

The transcriptomic enrichment results and STRING-based functional-association analyses suggested the involvement of TGF-β/BMP-Smad-related signaling in the chondrocyte response to IL-1β and eugenol. Because chondrocyte TGF-β/BMP-related signaling outcomes are partly influenced by the relative ALK1/ALK5 receptor profile, we next examined ALK1 and ALK5 expression and downstream Smad phosphorylation.

**FIGURE 5:**
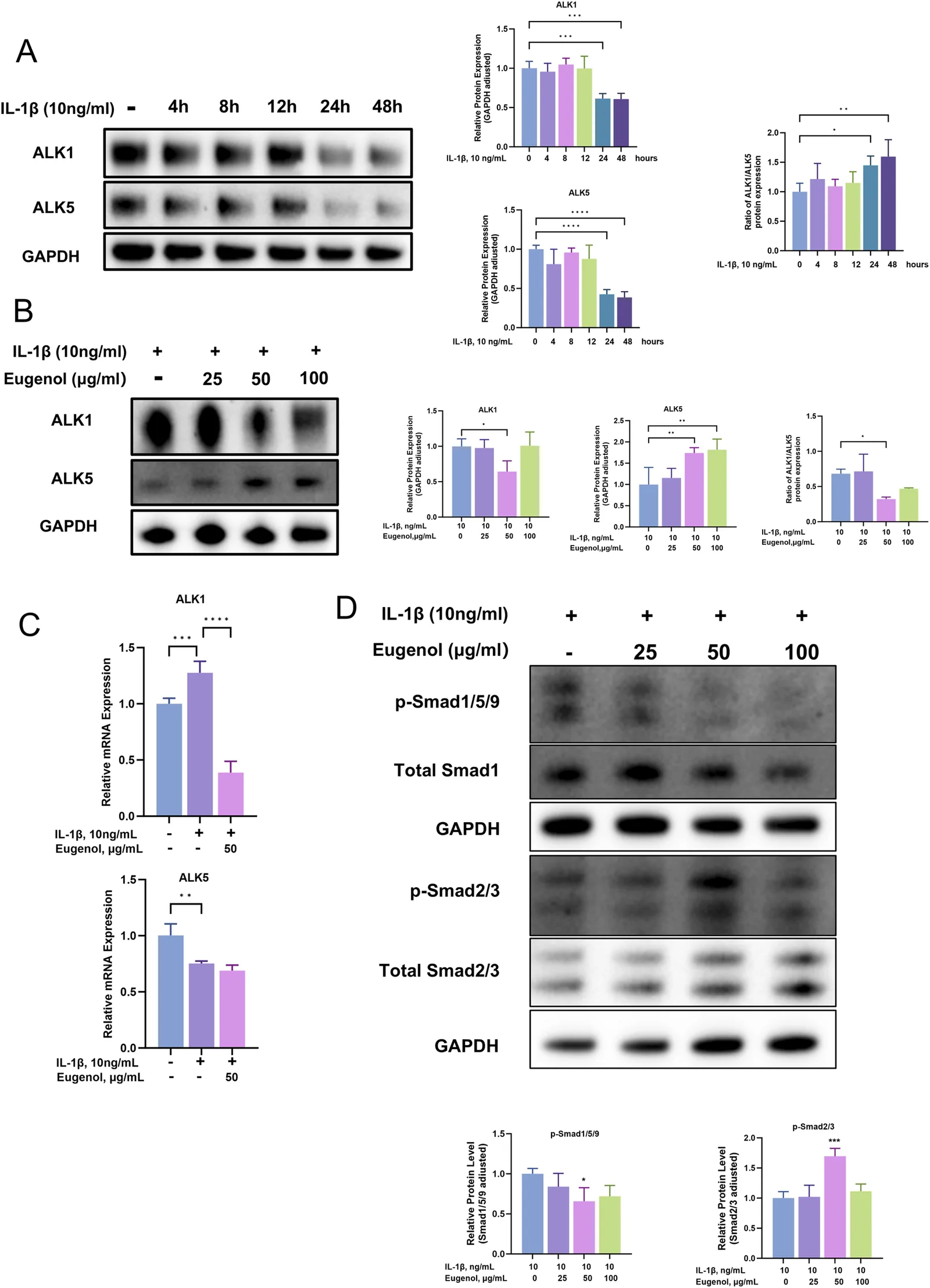
Eugenol attenuates the IL-1β-induced ALK1/ALK5 imbalance and shifts downstream Smad signaling in primary human chondrocytes. **(A)** Western blot analysis and densitometric quantification of ALK1 and ALK5 protein expression in primary human chondrocytes exposed to IL-1β for the indicated durations. The ALK1/ALK5 ratio was calculated to assess receptor-balance changes during inflammatory stimulation. **(B)** Western blot analysis and densitometric quantification showing the effects of eugenol pretreatment at 25, 50, and 100 μg/mL on IL-1β-induced changes in ALK1 and ALK5 protein expression and the ALK1/ALK5 ratio. **(C)** RT-qPCR analysis of *ACVRL1* (ALK1) and *TGFBR1* (ALK5) mRNA expression after IL-1β stimulation with or without eugenol treatment. **(D)** Western blot analysis of downstream Smad phosphorylation, including p-Smad1/5/9 and p-Smad2/3, under IL-1β stimulation with or without eugenol treatment. Quantification was performed relative to corresponding total Smad proteins, as appropriate. Data are presented as mean ± SD. Statistical comparisons are indicated in the figure.

Primary human chondrocytes were first exposed to IL-1β for the indicated durations to evaluate the time-dependent effects of inflammatory stimulation on ALK1 and ALK5 protein expression. Short-term IL-1β stimulation for 4–12 h did not markedly alter ALK1 or ALK5 expression compared with untreated controls. In contrast, prolonged IL-1β exposure reduced both ALK1 and ALK5 protein abundance, particularly at 24 and 48 h. Because ALK5 declined more markedly than ALK1, the normalized ALK1/ALK5 ratio increased at later stages of IL-1β stimulation (Figure 5A). This ratio reflects a relative receptor-expression profile and does not by itself establish ligand-specific receptor activity.

We next examined whether eugenol could counteract this IL-1β-induced receptor imbalance. Chondrocytes were pretreated with eugenol at 25, 50, or 100 μg/mL before IL-1β stimulation. Western blot analysis showed that eugenol differentially regulated ALK1 and ALK5 protein expression under inflammatory stimulation. Among the tested concentrations, 50 μg/mL eugenol produced the most pronounced reduction in the elevated ALK1/ALK5 ratio (Figure 5B). This effect was accompanied by decreased ALK1 protein abundance and partial restoration of ALK5 protein expression relative to the IL-1β group.

To determine whether these receptor changes were accompanied by transcriptional regulation, RT-qPCR analysis was performed for *ACVRL1*, which encodes ALK1, and *TGFBR1*, which encodes ALK5. IL-1β stimulation increased *ACVRL1* (ALK1) mRNA expression, whereas eugenol significantly attenuated this IL-1β-induced increase (Figure 5C). In contrast, IL-1β reduced *TGFBR1* (ALK5) mRNA expression, and eugenol did not significantly restore *TGFBR1* (ALK5) transcript levels compared with the IL-1β group. These results suggest that eugenol-associated reduction in the ALK1/ALK5 ratio was closely linked to transcriptional suppression of *ACVRL1* (ALK1), whereas the recovery of ALK5 protein was not paralleled by a detectable increase in *TGFBR1* (ALK5) mRNA.

We then investigated whether these receptor-expression changes were accompanied by changes in downstream Smad phosphorylation. Because ALK1 preferentially activates the Smad1/5/9 pathway, whereas ALK5 primarily signals through Smad2/3, phosphorylation levels of these Smad proteins were examined. Western blot analysis showed that eugenol, particularly at 50 μg/mL, was associated with reduced p-Smad1/5/9 levels and increased p-Smad2/3 levels under IL-1β stimulation (Figure 5D). Together with the receptor-expression data, these results indicate that eugenol treatment was associated with a lower ALK1/ALK5 ratio, reduced ALK1 abundance, partial recovery of ALK5 protein expression, lower p-Smad1/5/9 signal, and higher p-Smad2/3 signal under inflammatory stimulation.

Collectively, these results indicate that prolonged IL-1β exposure altered the ALK1/ALK5 receptor-expression profile, mainly through a more pronounced reduction in ALK5 protein. Eugenol, especially at 50 μg/mL, was associated with partial normalization of this receptor profile and reciprocal changes in Smad1/5/9 and Smad2/3 phosphorylation. However, changes in receptor abundance and Smad phosphorylation alone do not establish ligand-specific receptor activity or prove that the ALK1/ALK5-associated Smad axis is functionally required for the ECM-regulatory effects of eugenol. Therefore, siRNA-mediated knockdown of ALK1 and ALK5 was performed to determine the functional contribution of this signaling axis.

### 3.6 ALK1 and ALK5 depletion differentially modifies eugenol-associated ECM-marker responses

To determine the functional contribution of ALK1 and ALK5 to eugenol-responsive ECM regulation, siRNA-mediated loss-of-function experiments were performed in primary human chondrocytes. Cells were transfected with non-targeting control siRNA (si-NC), ALK1-specific siRNA (si-ALK1), or ALK5-specific siRNA (si-ALK5), followed by IL-1β stimulation in the presence or absence of eugenol.

**FIGURE 6:**
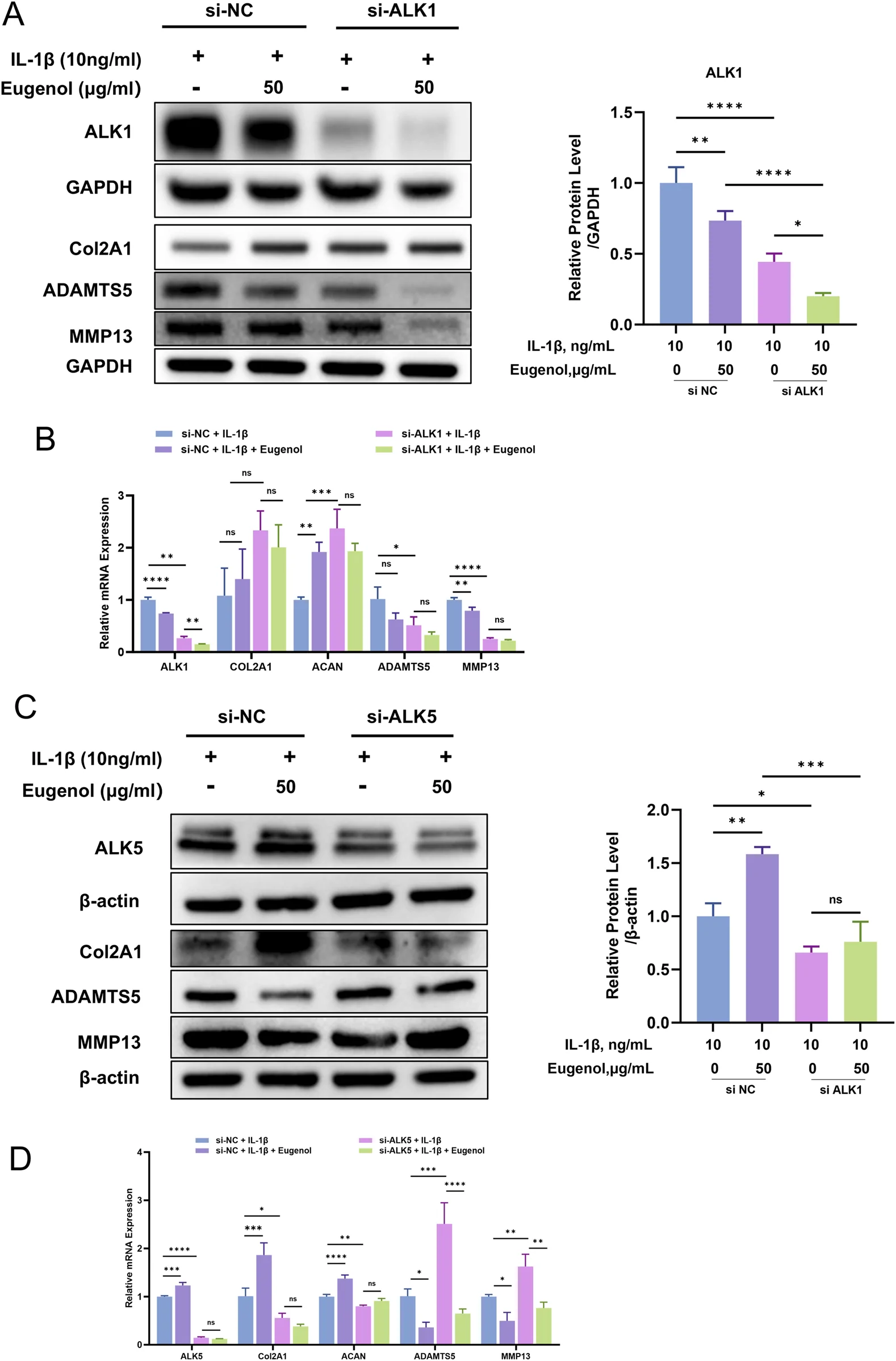
ALK1 and ALK5 knockdown differentially modify eugenol-associated ECM-marker responses in IL-1β-stimulated primary human chondrocytes. **(A,B)** Validation of ALK1 knockdown and assessment of ALK1-dependent effects of eugenol on ECM-related markers. ALK1 depletion partially phenocopied selected matrix-preserving and anti-catabolic effects of eugenol. **(C,D)** Validation of ALK5 knockdown and assessment of ALK5-dependent effects of eugenol on ECM-related markers. ALK5 depletion weakened several eugenol-associated matrix-restorative and anti-catabolic responses. Data are presented as mean ± SD. Statistical comparisons are indicated in the figure.

ALK1 knockdown was confirmed at both the protein and mRNA levels by Western blotting and RT-qPCR, respectively (Figures 6A,B). In si-NC-transfected cells exposed to IL-1β, eugenol significantly reduced ALK1 expression. In si-ALK1-transfected cells, ALK1 protein and transcript levels were markedly reduced, and the additional decrease associated with eugenol treatment was correspondingly smaller. These results confirmed efficient ALK1 depletion and supported the involvement of ALK1 in eugenol-associated regulation under inflammatory conditions.

In si-NC-transfected cells, eugenol increased COL2A1 and ACAN expression and reduced ADAMTS5 and MMP13 expression relative to IL-1β stimulation alone (Figures 6A,B). Under IL-1β-stimulated conditions, ALK1 knockdown alone produced changes in the same direction, including increased COL2A1 and ACAN expression and decreased ADAMTS5 and MMP13 expression, thereby partially phenocopying the ECM-protective effects of eugenol. Following ALK1 depletion, the additional effects of eugenol were less pronounced for several markers, although the magnitude and statistical support of these changes varied among the individual endpoints, and some responses were retained. These findings support a contribution of ALK1 to eugenol-responsive ECM regulation but indicate that ALK1 is unlikely to be the sole mediator of these effects (Figures 6A,B).

We therefore further evaluated the contribution of ALK5 using ALK5-specific siRNA. Efficient ALK5 knockdown was confirmed by reduced ALK5 protein and mRNA levels (Figures 6C,D). In si-NC-transfected cells, ALK5 expression was higher after eugenol treatment than after IL-1β stimulation alone, whereas this eugenol-associated increase was largely absent following ALK5 depletion. At the protein level, eugenol increased COL2A1 abundance in si-NC-transfected cells, but this response was substantially weakened after ALK5 knockdown (Figure 6C). Consistent with this observation, ALK5 depletion attenuated the eugenol-associated increases in COL2A1 and ACAN expression and reduced the ability of eugenol to suppress ADAMTS5 and MMP13 expression in IL-1β-stimulated cells (Figures 6C,D). Nevertheless, residual eugenol responses remained for some markers, indicating that the contribution of ALK5 was substantial but not exclusive.

Taken together, and considered alongside the Smad phosphorylation changes described above, these loss-of-function experiments support distinct but partial contributions of ALK1 and ALK5 to eugenol-responsive ECM regulation. ALK1 depletion partially phenocopied the matrix-preserving and anti-catabolic effects of eugenol and reduced selected additional responses to eugenol, whereas ALK5 depletion compromised several matrix-restorative and anti-catabolic responses. The persistence of some eugenol effects after either receptor was depleted suggests that additional signaling mechanisms may also contribute. Given this evidence for receptor involvement, we next performed exploratory computational and mutational analyses to examine whether a putative ALK1 region might be associated with eugenol responsiveness.

### 3.7 Exploratory mutagenesis analysis of the ALK1 Tyr279/His280 region

**FIGURE 7:**
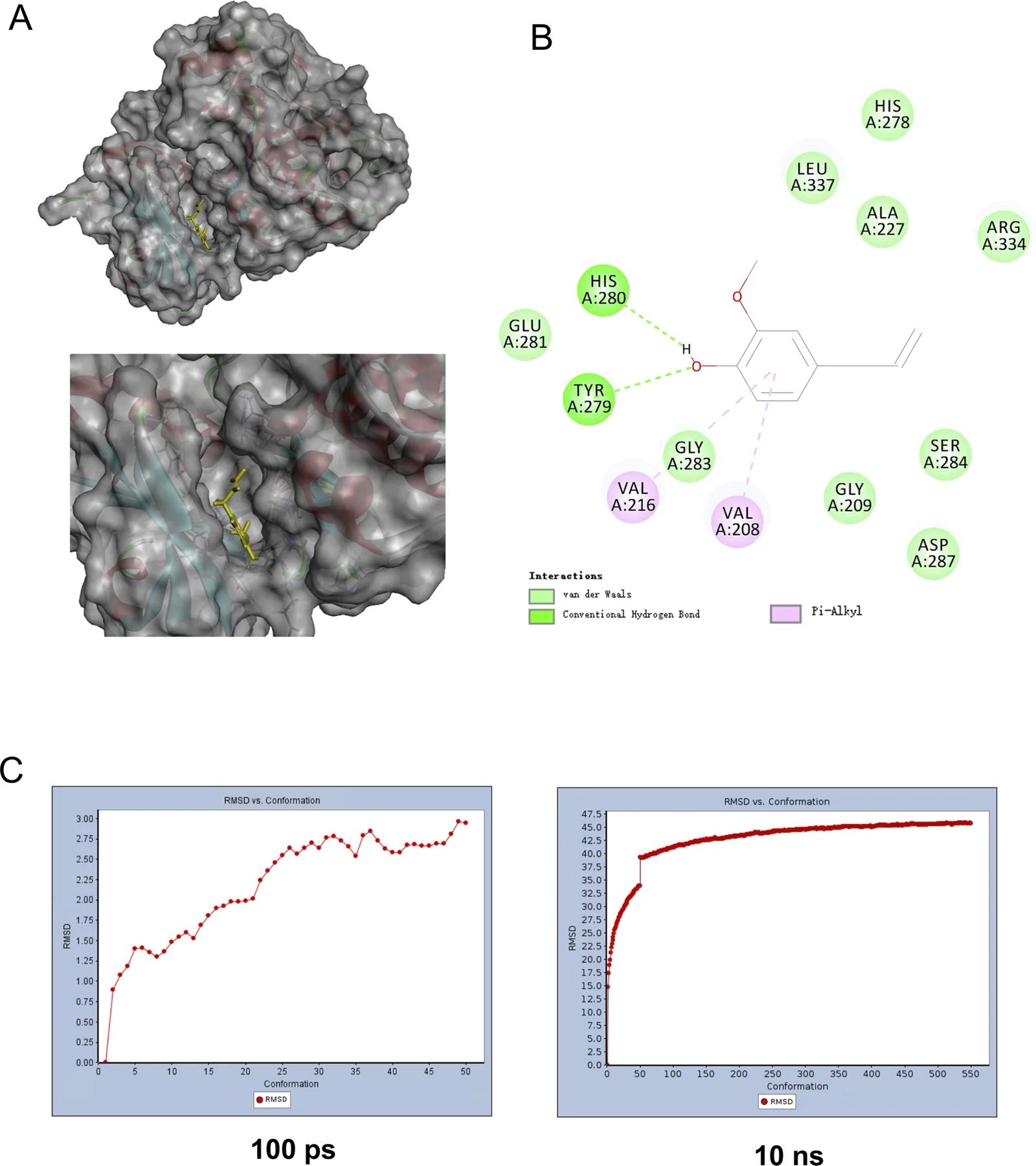
Molecular docking and molecular dynamics analyses of the predicted eugenol-ALK1 interaction. **(A)** Three-dimensional docking pose showing the predicted accommodation of eugenol within a local region of ALK1. **(B)** Two-dimensional interaction diagram showing predicted contacts between eugenol and ALK1 residues. Tyr279 and His280 were predicted to be in close proximity to eugenol, whereas His278, Glu281, Gly209, Ser284, Asp287, Ala227, Arg334, and Leu337 surrounded the local docking environment. **(C)** RMSD trajectories of the predicted eugenol-ALK1 complex during molecular dynamics simulations. The left panel shows the initial 100-ps trajectory, indicating early conformational adjustment of the docked complex. The right panel shows the 10-ns trajectory, showing an initial adjustment followed by relatively stable fluctuations. Note: These analyses represent computational predictions only.

To explore a potential structural basis for the association between eugenol treatment and ALK1-related signaling, molecular docking was performed as a hypothesis-generating computational analysis. The three-dimensional docking model predicted that eugenol could be accommodated within a local region on the surface of ALK1 (Figure 7A). The two-dimensional interaction diagram indicated predicted close contacts between eugenol and ALK1 residues Tyr279 and His280. Several neighboring residues, including His278, Glu281, Gly209, Ser284, Asp287, Ala227, Arg334, and Leu337, were located around the predicted local interaction environment (Figure 7B).

To further evaluate the dynamic behavior of the predicted eugenol-ALK1 docking pose, short-timescale molecular dynamics (MD) simulations were performed. The initial 100-ps trajectory showed early conformational adjustment of the predicted complex, indicating relaxation of the docking pose under the simulation conditions (Figure 7C, left panel). In the 10-ns simulation, the RMSD profile showed an initial adjustment followed by relatively stable fluctuations, suggesting that the predicted eugenol–ALK1 pose was dynamically plausible over the simulated period (Figure 7C, right panel). However, these short MD simulations support only the dynamic plausibility of the predicted docking pose and do not establish direct ligand–protein binding, cellular target engagement, or ALK1 as a confirmed molecular target of eugenol. Collectively, the docking and MD simulations results provide preliminary computational evidence suggesting potential structural compatibility between eugenol and an ALK1 region involving residues around Tyr279 and His280. These findings should be interpreted as hypothesis-generating evidence only. Direct binding or cellular target engagement between eugenol and ALK1 remains to be validated using CETSA, SPR, BLI, ITC, or other biochemical or biophysical assays. To investigate whether the docking-predicted ALK1 region encompassing Tyr279 and His280 contributed to the cellular response to eugenol, primary human chondrocytes were transiently transfected with empty vector, wild-type ALK1 (ALK1-WT), or the ALK1-Y279A/H280A double mutant (ALK1-Y279A/H280A). The cells were subsequently stimulated with IL-1β in the presence or absence of eugenol.

**FIGURE 8:**
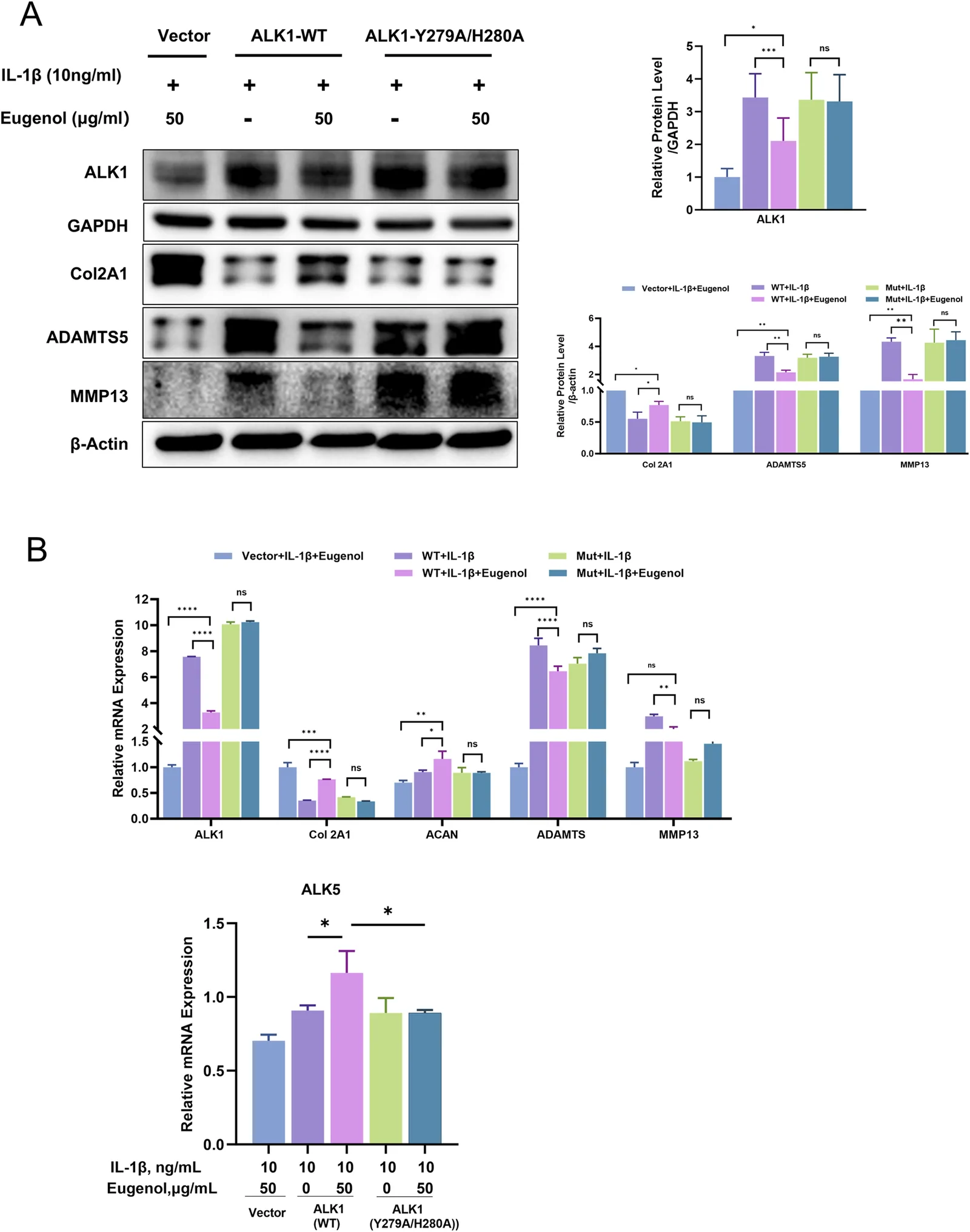
Effects of the ALK1-Y279A/H280A mutation on eugenol-associated responses in IL-1β-stimulated chondrocytes. Primary human chondrocytes were transiently transfected with empty vector, wild-type ALK1 (ALK1-WT), or the ALK1-Y279A/H280A mutant and were subsequently stimulated with IL-1β in the presence or absence of eugenol. **(A)** Western blotting and densitometric quantification of ALK1 and ECM-related proteins, as indicated. **(B)** RT-qPCR analysis of *ALK1/ALK5* and ECM-related genes, as indicated. Data are presented as mean ± SD. Statistical comparisons are indicated in the figure.

Western blotting confirmed increased ALK1 protein abundance in cells transfected with either ALK1-WT or ALK1-Y279A/H280A compared with empty-vector controls (Figure 8A). Under IL-1β stimulation, eugenol treatment was associated with reduced ALK1 protein abundance in ALK1-WT-transfected cells. This reduction was less pronounced in cells expressing the Y279A/H280A mutant, in which ALK1 abundance remained comparatively high after eugenol treatment. However, direct comparisons of eugenol responsiveness between the wild-type and mutant constructs did not consistently reach statistical significance. Therefore, evidence for a mutation-dependent effect on ALK1 abundance should be considered preliminary (Figure 8A). We next examined ECM-related markers. In ALK1-WT-transfected cells, eugenol reduced IL-1β-associated ADAMTS5 and MMP13 protein and mRNA expression. The corresponding reductions were less pronounced in cells expressing the ALK1-Y279A/H280A mutant, although the magnitude of this difference varied between assays (Figures 8A,B). Eugenol-associated restoration of COL2A1 and suppression of MMP13 were also attenuated in selected comparisons involving the mutant construct, whereas the effects on ACAN were less consistent. These marker-dependent patterns suggest that disruption of the Tyr279/His280 region may alter selected ECM-related responses to eugenol rather than uniformly abolishing eugenol responsiveness. ALK5 mRNA expression was also assessed. In ALK1-WT-transfected cells, eugenol treatment was associated with increased ALK5 transcript abundance under IL-1β stimulation. These findings were consistent with a possible contribution of the Tyr279/His280 region to selected eugenol-associated responses, although the limited statistical support for direct wild-type-versus-mutant comparisons warrants cautious interpretation (Figure 8B). Nevertheless, the modest magnitude of this effect and the limited statistical support for direct wild-type-versus-mutant comparisons warrant cautious interpretation.

Collectively, the Y279A/H280A substitutions were associated with altered eugenol responsiveness at the levels of ALK1 abundance, selected ECM-related markers, and ALK5 transcription. However, these effects were marker-dependent, and direct comparisons between the wild-type and mutant constructs were not consistently significant. Moreover, the substitutions may have altered receptor conformation, stability, abundance, trafficking, localization, or signaling competence. Therefore, these results provide preliminary evidence for the functional relevance of the Tyr279/His280 region but neither establish this region as a ligand-binding site nor demonstrate direct physical engagement between eugenol and ALK1.

### 3.8 Juvenile-versus-aged and ACLT-associated ALK1/ALK5 receptor-profile changes in cartilage

Because the structural and histological protective effects of eugenol in an ACLT-induced OA model were reported previously (Wu et al., 2023), the present *in vivo* experiments focused on whether eugenol modulates the ALK1/ALK5 receptor- level imbalance in articular cartilage.

**FIGURE 9:**
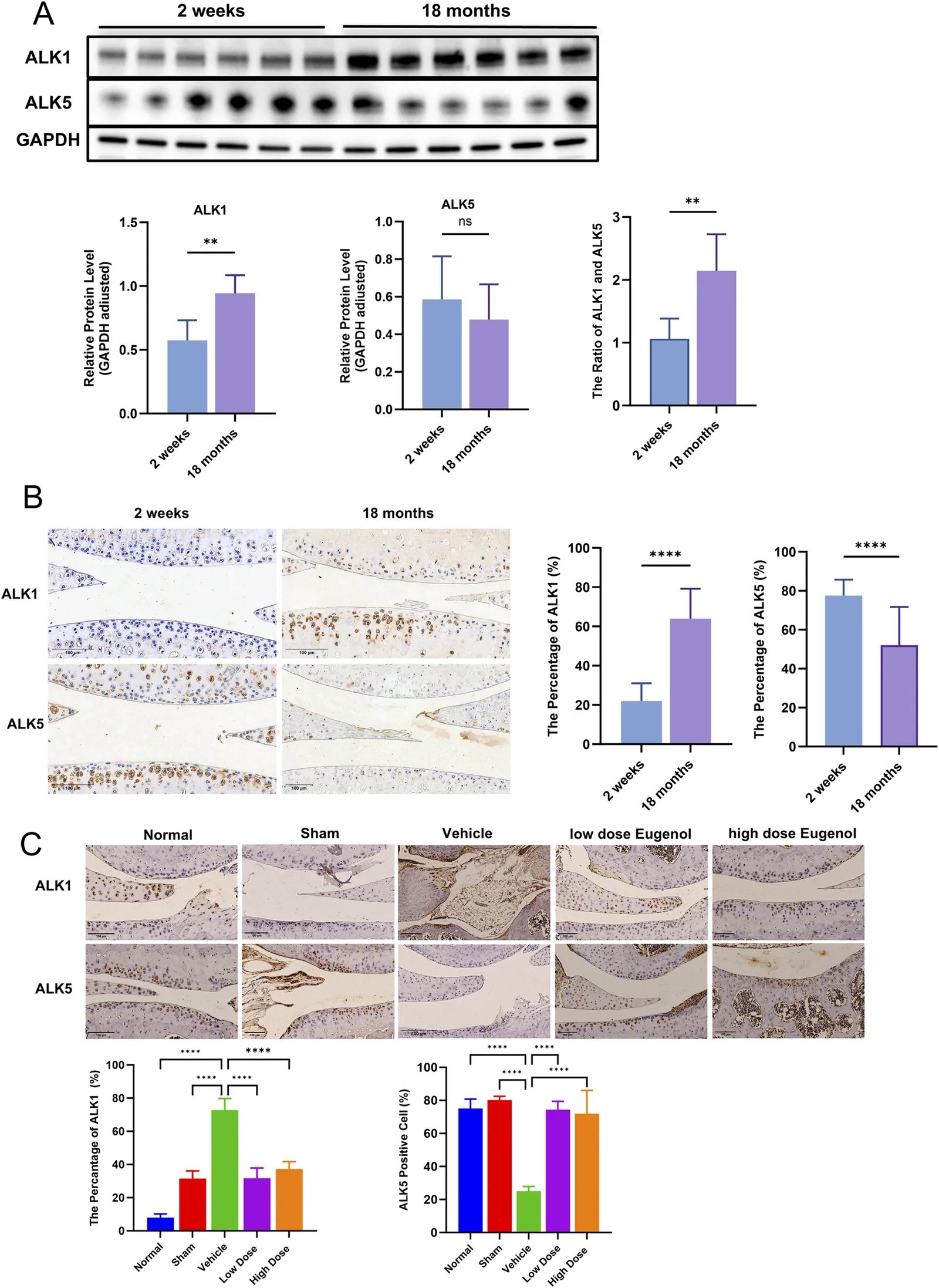
Juvenile-versus-aged and ACLT-associated changes in the ALK1/ALK5 receptor profile in cartilage and their modulation by eugenol. **(A)** Western blot analysis and densitometric quantification of ALK1 and ALK5 protein expression in knee articular cartilage from 2-week-old and 18-month-old C57BL/6 mice. The ALK1/ALK5 protein ratio was calculated to evaluate receptor-balance changes. **(B)** Representative immunohistochemical staining and quantification of ALK1- and ALK5-positive chondrocytes in juvenile and aged articular cartilage. Scale bar = 100 μm. **(C)** Representative immunohistochemical staining and quantification of ALK1- and ALK5-positive chondrocytes in articular cartilage from control, sham, ACLT + vehicle. ACLT + low-dose eugenol, and ACLT + high-dose eugenol groups. Scale bar = 100 μm. Data are presented as mean ± SD. Statistical comparisons are indicated in the figure.

To determine whether the ALK1/ALK5 receptor imbalance observed *in vitro* was also detectable in articular cartilage *in vivo*, we first compared ALK1 and ALK5 expression in knee cartilage from 2-week-old and 18-month-old C57BL/6 mice. Western blot analysis showed that ALK1 protein expression was markedly increased in aged cartilage, whereas total ALK5 protein expression was not significantly different between the two age groups. Consequently, the ALK1/ALK5 protein ratio was significantly elevated in aged cartilage (Figure 9A). These findings indicate a juvenile-versus-aged difference characterized by a higher relative ALK1/ALK5 receptor profile in 18-month-old cartilage.

Immunohistochemical staining provided further tissue-level information on ALK1 and ALK5 distribution. Compared with juvenile cartilage, aged cartilage exhibited a higher percentage of ALK1-positive chondrocytes and a lower percentage of ALK5-positive chondrocytes (Figure 9B). Although total ALK5 protein abundance was not significantly altered by Western blotting, IHC suggested an altered cellular distribution or a reduced ALK5-positive chondrocyte frequency in aged cartilage. These tissue-level findings are consistent with an ALK1-skewed receptor profile in 18-month-old cartilage, although developmental-stage differences and aging-related changes cannot be fully separated in this comparison.

We next evaluated whether eugenol could attenuate this receptor imbalance in the ACLT-induced mouse model. In vehicle-treated ACLT mice, articular cartilage showed increased ALK1 immunoreactivity and reduced ALK5 immunoreactivity compared with the normal and sham groups, indicating that surgically induced OA was associated with an ALK1-dominant receptor profile. In contrast, eugenol treatment reduced the proportion of ALK1-positive chondrocytes and preserved the proportion of ALK5-positive chondrocytes in ACLT cartilage (Figure 9C). Both low- and high-dose eugenol showed receptor-modulating effects, with no statistically significant difference between the two doses.

Collectively, these *in vivo* findings indicate that the ALK1/ALK5 receptor profile differs between juvenile and aged cartilage and is also altered in ACLT-induced OA cartilage. Eugenol partially normalized the ACLT-associated receptor profile change by reducing ALK1 immunoreactivity and maintaining a higher percentage of ALK5-positive chondrocytes relative to vehicle-treated ACLT cartilage. These receptor-level data are consistent with the *in vitro* ALK1/ALK5-associated Smad findings but should not be interpreted as independent structural histological evidence of cartilage protection in the current cohort. No significant differences in initial or final body weight were observed among the ACLT + vehicle, ACLT + low-dose eugenol, and ACLT + high-dose eugenol groups (Table 4), suggesting no overt body-weight-associated intolerance under the present treatment regimen.

**TABLE 4:** Initial and final body weights during the ACLT experiment.

Group	Initial body weight (g)	Final body weight (g)
ACLT + vehicle	26.14 ± 0.96	28.98 ± 0.92
ACLT + eugenol, 5 mg/kg	26.50 ± 1.01	29.18 ± 1.18
ACLT + eugenol, 10 mg/kg	26.26 ± 0.93	28.92 ± 0.76

Data are presented as mean ± SD. Comparisons among groups were performed using one-way ANOVA. For initial body weight, F = 0.040 and P = 0.961. For final body weight, F = 0.311 and P = 0.739. No significant differences in initial or final body weight were observed among the ACLT + vehicle, ACLT + low-dose eugenol, and ACLT + high-dose eugenol groups.

## 4 Discussion

Osteoarthritis is increasingly recognized as a disorder of disrupted cartilage homeostasis, in which persistent inflammatory, mechanical, and age-associated stressors drive chondrocytes from a stable matrix-preserving phenotype toward a catabolic, hypertrophic-like, and inflammatory state (Abramoff and Caldera, 2020; Woodell-May and Sommerfeld, 2020; Sanchez-Lopez et al., 2022; Yao et al., 2023; Tang et al., 2025). In the present study, eugenol attenuated IL-1β-associated dysregulation of ECM-related markers in primary human chondrocytes. Eugenol increased ACAN, COL2A1, and SOX9 expression, reduced MMP13, ADAMTS5, COL10A1, and RUNX2 expression, and attenuated IL-6-associated inflammatory activation. These coordinated molecular changes are consistent with partial preservation of a matrix-supportive chondrocyte phenotype under inflammatory stress. Mechanistically, eugenol was associated with partial normalization of the IL-1β-altered ALK1/ALK5 receptor-expression profile and reciprocal changes in Smad1/5/9 and Smad2/3 phosphorylation. Together with the loss-of-function experiments, these findings support a partial contribution of ALK1/ALK5-associated Smad signaling to eugenol-responsive ECM regulation.

Among the ECM-related changes observed in this study, restoration of COL2A1 is particularly important. COL2A1 encodes type II collagen, the predominant collagenous component of hyaline cartilage, and its preservation is a key indicator of cartilage matrix integrity. Therefore, the recovery of COL2A1 expression by eugenol at both the mRNA and protein levels suggests that eugenol supports matrix preservation rather than merely suppressing inflammatory mediators. In parallel, inhibition of MMP13 is mechanistically relevant because MMP13 directly mediates type II collagen degradation and has been linked to an increased ALK1/ALK5 receptor profile and ALK1-associated catabolic signaling in OA cartilage (Blaney Davidson et al., 2009; Little and Fosang, 2010). Beyond these core matrix readouts, eugenol restored SOX9 expression and suppressed COL10A1 and RUNX2 under IL-1β stimulation, suggesting preservation of the chondrocytic phenotype and attenuation of hypertrophic-like remodeling. These transcriptional findings, together with protein-level and immunofluorescence evidence, strengthen the conclusion that eugenol attenuates inflammatory-catabolic ECM dysregulation in chondrocytes.

Transcriptomic analyses provided broader molecular context for the eugenol response. Because the RNA-seq groups represented discrete treatment conditions rather than true temporal sampling points, direct pairwise differential-expression comparisons were used as the primary analytical strategy. This analysis identified 2,344 IL-1β-responsive genes, among which 880 and 973 genes were directionally counter-regulated by 50 and 100 μg/mL eugenol, respectively, with 840 genes commonly counter-regulated by both eugenol doses. It should be emphasized that these gene lists were generated using exploratory screening criteria based on nominal P value and fold-change thresholds, whereas Benjamini–Hochberg-adjusted P values were reported for transparency. Therefore, the transcriptomic findings should be interpreted as hypothesis-generating rather than as stand-alone confirmatory evidence. In addition, the term “counter-regulated” denotes an opposite direction of differential expression relative to the IL-1β response, but does not necessarily indicate complete normalization to the untreated control state. Functional enrichment analysis of commonly counter-regulated genes suggested that eugenol affects a broad inflammatory-catabolic regulatory network in IL-1β-stimulated chondrocytes. Enriched biological processes included immune and inflammatory responses, cellular response to IL-1, cell migration and adhesion, proliferation, cell-cycle regulation, and signal transduction. KEGG analysis further highlighted JAK-STAT, TNF, MAPK, NF-κB, AMPK, HIF-1, PI3K-Akt, Wnt, VEGF, and TGF-β/BMP-related signaling pathways. These results suggest that ALK1/ALK5-associated Smad signaling represents one functionally implicated component of a broader eugenol-responsive network rather than the sole mechanism underlying the observed chondroprotective effects. Because RNA-seq was performed at 24 h whereas most phenotypic and protein-level experiments were performed at 48 h, the transcriptomic data should also be interpreted as earlier treatment-associated molecular changes rather than a same-time-point explanation of all downstream phenotypes.

Mfuzz clustering and STRING-based analyses were used as supportive exploratory approaches. Because the four RNA-seq groups did not represent a true time series, Mfuzz clustering was not interpreted as a temporal trajectory and was not used as the primary basis for target prioritization. Similarly, STRING analysis contextualized ACVRL1/ALK1 within a TGF-β/BMP-Smad-related functional association network, but did not identify ACVRL1 as a direct molecular target of eugenol. ACVRL1/ALK1 was further investigated primarily because of its established biological relevance to OA-associated TGF-β/BMP-Smad signaling, its relationship to MMP13-associated matrix catabolism, and the receptor-level changes observed in the present experimental system.

TGF-β/BMP-related signaling has dual and context-dependent effects in cartilage. In healthy cartilage, ALK5-associated Smad2/3 signaling generally supports chondrocyte homeostasis and matrix synthesis, whereas increased ALK1-associated Smad1/5/9 signaling has been associated with hypertrophy, matrix catabolism, and OA progression (Blaney Davidson et al., 2009; Blaney Davidson et al., 2015; Finnson et al., 2010; van der Kraan, 2017; Thielen et al., 2019; Thielen et al., 2023; Moon et al., 2026). The study by Blaney Davidson et al. is particularly relevant because it directly linked an increased ALK1/ALK5 ratio to elevated MMP13 expression in human and mouse OA cartilage (Blaney Davidson et al., 2009). In the present study, prolonged IL-1β exposure reduced both ALK1 and ALK5 protein expression, but ALK5 declined more prominently, resulting in an increased ALK1/ALK5 ratio. This ratio reflects a relative receptor-expression profile and does not by itself establish ligand-specific receptor activity.

Eugenol, particularly at 50 μg/mL, was associated with a lower ALK1/ALK5 ratio, reduced ALK1 abundance, partial restoration of ALK5 protein expression, reduced Smad1/5/9 phosphorylation, and increased Smad2/3 phosphorylation under IL-1β stimulation. Notably, receptor-level and transcriptional changes were not completely parallel. IL-1β increased ACVRL1 mRNA expression, whereas prolonged IL-1β exposure reduced total ALK1 protein abundance; similarly, eugenol partially recovered ALK5 protein levels without significantly restoring TGFBR1 mRNA expression. These observations are compatible with post-transcriptional, translational, protein-stability, receptor-trafficking, or degradation-related regulation, but these mechanisms were not directly tested. In addition, ALK1 can participate in BMP-related signaling, and Smad1/5/9 phosphorylation can be regulated by multiple type-I receptors. Therefore, the present data do not establish that eugenol selectively modifies ligand-specific TGF-β signaling.

Loss-of-function experiments further supported, but did not prove exclusivity of, the ALK1/ALK5-associated mechanism. ALK1 depletion partially phenocopied selected anti-catabolic and matrix-preserving effects of eugenol and reduced additional responses to eugenol for several ECM-related markers. Conversely, ALK5 depletion weakened several matrix-restorative and anti-catabolic responses to eugenol, including effects on ACAN, COL2A1, MMP13, and ADAMTS5. These findings suggest that suppression of ALK1-associated signaling may contribute to the anti-catabolic component of the eugenol response, whereas preservation of ALK5-associated signaling may be important for the full matrix-supportive effect. However, residual eugenol responsiveness after either ALK1 or ALK5 depletion indicates that additional pathways are likely involved. The observation that 50 μg/mL eugenol produced the most pronounced receptor-profile modulation, whereas a higher concentration did not uniformly enhance this effect, also suggests a potentially non-linear or biphasic pharmacological response. Future dose-response studies with broader concentration ranges and pathway-specific readouts will be needed to define the optimal therapeutic window.

This pathway-oriented interpretation is consistent with the broader concept that modulation of defined receptor-associated signaling modules can protect joint tissues under OA-related stress. For example, Fu et al. demonstrated that 14-3-3ε functions as an intracellular component of the TNFR2 receptor complex and that activation of this axis protects against OA by regulating inflammatory and catabolic responses (Fu et al., 2021). Although the TNFR2/14-3-3ε axis is distinct from the ALK1/ALK5-associated Smad signaling examined here, both studies support the concept that receptor-proximal signaling balance can be an important determinant of chondrocyte behavior during OA-related stress.

The involvement of Smad-related signaling in musculoskeletal responses to bioactive compounds is also supported by studies outside the cartilage field. Dong et al. reported that higenamine promotes osteogenesis through IQGAP1/SMAD4 signaling and prevents age- and estrogen-dependent bone loss in mice, whereas ellagic acid promotes osteoblast differentiation by activating SMAD2/3 signaling and alleviates bone mass loss in ovariectomized mice (Dong et al., 2023; Dong et al., 2024). These studies focused mainly on osteoblast-lineage cells and bone phenotypes rather than chondrocytes and therefore cannot be directly extrapolated to OA cartilage. Nevertheless, they support the broader principle that natural compounds can influence musculoskeletal tissue homeostasis through Smad-associated signaling networks. In this context, the present study extends this concept to inflammatory chondrocytes by implicating ALK1/ALK5-associated Smad signaling in the ECM-regulatory response to eugenol.

The computational and mutational analyses should be interpreted cautiously. Molecular docking predicted that eugenol could be accommodated within a putative ALK1 region involving Tyr279 and His280, and short molecular-dynamics simulations supported the dynamic plausibility of this predicted docking pose. However, docking and short-timescale simulations are inherently hypothesis-generating and cannot prove direct ligand–protein binding or cellular target engagement. Similarly, the Y279A/H280A mutagenesis data may reflect altered receptor conformation, abundance, stability, trafficking, localization, or downstream signaling competence rather than loss of direct ligand-contact capacity. Therefore, the relationship between eugenol and the Tyr279/His280 region of ALK1 should be regarded as computationally predicted and functionally implicated, but not biochemically validated as direct target engagement. Future studies should directly evaluate whether eugenol engages ALK1 using biochemical or biophysical approaches, including cellular thermal shift assays, surface plasmon resonance, biolayer interferometry, and isothermal titration calorimetry. siRNA-resistant rescue constructs and ligand-competition experiments may further clarify the functional contribution of ALK1 and the Tyr279/His280 region.

The *in vivo* findings provide receptor-level consistency with the cell-culture data but should not be overinterpreted as independent structural evidence of cartilage protection. Standardized histological assessment, including H&E staining, Safranin O/Fast Green staining, proteoglycan preservation, cartilage surface integrity assessment, and OARSI-based scoring, is essential for evaluating disease modification in experimental OA models (Pritzker et al., 2006; Glasson et al., 2010). In our previous ACLT-based eugenol study using a comparable experimental model and treatment regimen, eugenol alleviated cartilage surface damage, reduced proteoglycan loss, improved OARSI-based scores, and modulated ECM-related markers, including COL2A1, ACAN, MMP13, and ADAMTS5 (Wu et al., 2023). To avoid duplicate publication or inappropriate reuse of previously published histological images and scoring data, those structural histological and ECM-marker IHC results were not re-presented as newly generated data in the current manuscript. Instead, the present study extends our prior findings by focusing on receptor-level ALK1/ALK5 changes and downstream signaling mechanisms. In the present study, comparison between 2-week-old and 18-month-old mouse cartilage showed an increased ALK1/ALK5 protein ratio and altered ALK1- and ALK5-positive chondrocyte distribution in aged cartilage. Because this comparison involved juvenile and aged animals, developmental-stage differences and aging-related changes cannot be completely separated. Therefore, these findings should be interpreted as juvenile-versus-aged or maturation- and age-associated receptor-profile differences rather than definitive proof of aging alone. In the ACLT-induced OA model, vehicle-treated ACLT cartilage exhibited increased ALK1 immunoreactivity and reduced ALK5 immunoreactivity, consistent with an ALK1-skewed receptor profile. Eugenol treatment reduced ALK1-positive chondrocytes and preserved ALK5-positive chondrocytes in ACLT cartilage. These receptor-level observations support the relevance of ALK1/ALK5 imbalance in OA-like cartilage and are consistent with the *in vitro* ALK1/ALK5-Smad findings. However, future *in vivo* studies incorporating receptor expression, p-Smad1/5/9 and p-Smad2/3 staining, structural histology, and functional joint outcomes within the same animal cohort will be needed to determine whether receptor-profile modulation is associated with structural cartilage preservation.

The safety profile and pharmacokinetic properties of eugenol should be interpreted cautiously. *In vitro*, 50 μg/mL eugenol was selected based on our previously published CCK-8 data showing no significant reduction in chondrocyte viability after 48 h under comparable conditions (Wu et al., 2023). However, the present study did not perform CCK-8, Live/Dead, apoptosis assays, or equivalent cell-death assays under the exact vehicle exposure, culture conditions, and treatment duration used here. Therefore, although the selected concentration was supported by previous viability data, cytotoxicity under the precise current experimental conditions was not independently re-evaluated. This limitation is particularly relevant because transcriptomic and signaling responses can be influenced by sublethal cellular stress even in the absence of overt viability loss.

*In vivo*, eugenol was administered intraperitoneally using a vehicle containing DMSO, PEG300, Tween-80, and saline. No significant differences in initial or final body weight were observed among the ACLT + vehicle, ACLT + low-dose eugenol, and ACLT + high-dose eugenol groups, suggesting no overt body-weight-associated intolerance under the current dosing regimen. Nevertheless, body weight alone is insufficient to establish systemic safety. The present study did not include systematic gross-health scoring, serum biochemical indicators of hepatic and renal function, such as ALT, AST, BUN, and CREA, or H&E staining of major organs. In addition, because eugenol is rapidly metabolized *in vivo*, once-weekly intraperitoneal administration should not be assumed to maintain continuous effective concentrations in plasma, synovial fluid, or cartilage. Thus, the regimen used here should be interpreted as an efficacy-oriented intermittent exposure protocol adopted from our previous ACLT study rather than a pharmacokinetically optimized dosing strategy. Future studies incorporating pharmacokinetic/pharmacodynamic analysis, tissue-distribution measurements, dose-response testing, and extended biosafety evaluation will be required to optimize the dosing regimen and support translational development. From a translational perspective, future development of eugenol-based OA interventions may require optimized delivery strategies to improve local joint exposure while reducing systemic exposure. Recent advances in musculoskeletal biomaterials have shown that therapeutic platforms can be designed to integrate disease-control functions with tissue-regenerative capacity, as illustrated by dual-function biomaterials developed for tumor suppression and bone regeneration after osteosarcoma surgery (Dong et al., 2025). Although such biomaterial strategies are not directly equivalent to OA pharmacotherapy, they provide a useful conceptual framework for future local delivery or sustained-release approaches aimed at cartilage protection. For OA applications, intra-articular delivery systems, hydrogel-based sustained-release platforms, nanoparticle carriers, or cartilage-targeting formulations may help overcome the limitations associated with rapid systemic metabolism and intermittent systemic exposure of eugenol.

This study has several limitations. First, although ALK1 and ALK5 knockdown experiments provide functional evidence supporting the involvement of ALK1/ALK5-associated Smad signaling, they do not fully exclude alternative mechanisms. Multiple independent siRNAs, siRNA-resistant rescue constructs, receptor-specific rescue experiments, and genetic models would further strengthen causal inference. Second, direct target engagement between eugenol and ALK1 was not validated. Third, *in vitro* cytotoxicity assessment under the exact current experimental conditions and comprehensive *in vivo* biosafety evaluation were not performed. Fourth, the newly generated *in vivo* data focused mainly on ALK1/ALK5 receptor expression and did not include structural histological endpoints in the same animal cohort. Finally, validation in human OA cartilage specimens and additional OA models would strengthen the translational relevance of these findings.

In conclusion, eugenol attenuated IL-1β-induced inflammatory-catabolic changes in primary human chondrocytes and improved the expression profile of multiple ECM-related, hypertrophy-associated, and inflammatory markers. The findings support a model in which modulation of ALK1/ALK5-associated Smad signaling partially contributes to eugenol-responsive ECM regulation. ALK1 depletion phenocopied selected anti-catabolic effects, whereas ALK5 depletion weakened several matrix-restorative responses, indicating distinct but nonexclusive roles for the two receptors. Computational docking, molecular-dynamics simulations, and mutagenesis analyses remain exploratory and do not establish ALK1 as a direct molecular target of eugenol. Future studies should evaluate direct target engagement, ligand-specific signaling, pharmacokinetics, safety, and the relationship between receptor modulation and structural cartilage outcomes in the same *in vivo* cohort.

## Data Availability

The data presented in the study are deposited in the CNSA repository, accession number CNP0010012.
